# Nanoradiosensitizer with good tissue penetration and enhances oral cancer radiotherapeutic effect

**DOI:** 10.1016/j.biomaterials.2022.121769

**Published:** 2022-09-01

**Authors:** Di Jing, Nian Jiang, Fengyi Wang, Chunping Mao, Shujun Han, Pui Yan Ho, Wenwu Xiao, Yuanpei Li, Jian Jian Li, Lu Zhang, Kit S. Lam

**Affiliations:** a Department of Biochemistry and Molecular Medicine, UC Davis Comprehensive Cancer Center, University of California Davis, Sacramento, CA, USA; b Department of Oncology, National Clinical Research Center for Geriatric Disorders, Xiangya Hospital, Central South University, Changsha, Hunan, China; c Department of Biomedical Engineering, Southern University of Science and Technology, Shenzhen, Guangdong, China; d Department of Radiation Oncology, School of Medicine, University of California Davis, Sacramento, CA, USA; e Department of Neurosurgery, Xiangya Hospital, Central South University, Changsha, China; f Division of Hematology and Oncology, Department of Internal Medicine, School of Medicine, University of California Davis, Sacramento, CA, USA

**Keywords:** Radio-sensitizer, Nano-micelle, Radiotherapy, Oral cancer

## Abstract

Low dose non-toxic disulfide cross-linked micelle (DCM) encapsulated paclitaxel (PTX) was found to be highly efficacious as a radiosensitizer against oral cancer preclinical model. Intensity-modulated radiation therapy was locally administered for three consecutive days 24 h after intravascular injection of DCM-[PTX] at 5 mg/kg PTX. DCM-[PTX] NPs combined with conventional radiotherapy (2 Gy) resulted in a 1.7-fold improvement in therapeutic efficacy compared to conventional PTX plus radiotherapy. Interestingly, we found that radiotherapy can decrease tight junctions and increase the accumulation of DCM-[PTX] in tumor sites. Stereotactic body radiotherapy (SBRT) given at 6 Gy was used to further investigate the synergistic anti-tumor effect. Tumor tissues were collected to analyze the relationship between the time interval after SBRT and the biodistribution of the nano-materials. Compared to combination DCM-[PTX] with conventional radiation dose, combination DCM-PTX with SBRT was found to be more efficacious in inhibiting tumor growth.

## 1. Introduction

Cancer of the oral cavity was diagnosed in over 350,000 individuals worldwide in 2018, accounting for 2% of all cancers and more than 170,000 patient deaths due to the disease. Oral cancer with high morbidity and mortality is mainly the result of the advanced clinical stage at the initial diagnosis [1]. Surgery is the standard treatment for oral cancer, but recurrence occurred in 45.6% of patients in the 1980s and 36.1% of patients from 2010 to 2017, after the development of improved surgical methods, with five-year overall survival rate increased from 60% to 70%, respectively [2]. Postoperative radiotherapy can improve the patient survival rate, and concurrent chemo-radiotherapy can achieve an even better treatment response [3]. However, many patients cannot complete the full course of chemo-radiation treatment because of severe toxicity such as acute oral mucositis, skin ulcers, systemic blood suppression and worsening of surgery-related wounds [4].

Nanomedicine as a promising treatment concept could improve the tumor targeting property and prolong the drug circulation time in blood, in part because of the increased stability of the encapsulated drug and the enhanced permeability and retention (EPR) effect of the nanoparticles (NPs) at the tumor sites [5,6]. A few years ago, we reported the development of a cholic acid based self-assembled and disulfide cross-linked polymeric micellar nanoplatform for convenient drug formulation with high loading and superior biodistribution *in vivo* [7]. Although other studies have utilized disulfide bonds to form cross-linked micelles encapsulating doxorubicin and methotrexate, these approaches require additional processing steps that affect drug loading efficiency, nanoparticle size, and stability during blood circulation [8,9]. Paclitaxel (PTX) has been approved by Federal Drug Administration (FDA) as the first-line standard chemotherapeutic agent against head and neck, lung, breast, and ovarian cancers [10,11]. We have obtained strong evidence on the efficacy of disulfide cross-linked micelles (DCM) loaded with PTX, which exhibit minimum systemic toxicity and produce excellent tumor responses in xenograft models compared with Abraxane and Taxol [7]. Clinical trials demonstrated that breast cancer patients treated with PTX every week showed improved disease-free and overall survival rates compared with patients treated with PTX every three weeks [12]. Pre-surgery chemo-radiotherapy of esophageal cancer patients resulted in a 35% decreased risk of death. PTX binding to tubulin inhibits the function of microtubules and reduces cell proliferation in the G_2_/M phases of the cell cycle, which is the most radiosensitive phase [13,14]. Although radiotherapy with concurrent chemotherapy can significantly improve the prognosis of head and neck cancers, severe oral mucositis limits its widespread use in oral cancer. Majority of patients cannot tolerate the complications, such as pain, poor nutrition and systemic myelosuppression [15]. We have previously demonstrated the high efficacy and low toxicity of DCM-[PTX] NPs at 30 mg/kg PTX in ovarian cancer xenograft model [7]. DCM-[PTX] NPs given at low dose (5 mg/kg PTX) is expected to be totally non-toxic but can greatly synergize the radiotherapeutic effects in oral cancer.

**Fig. 1.**
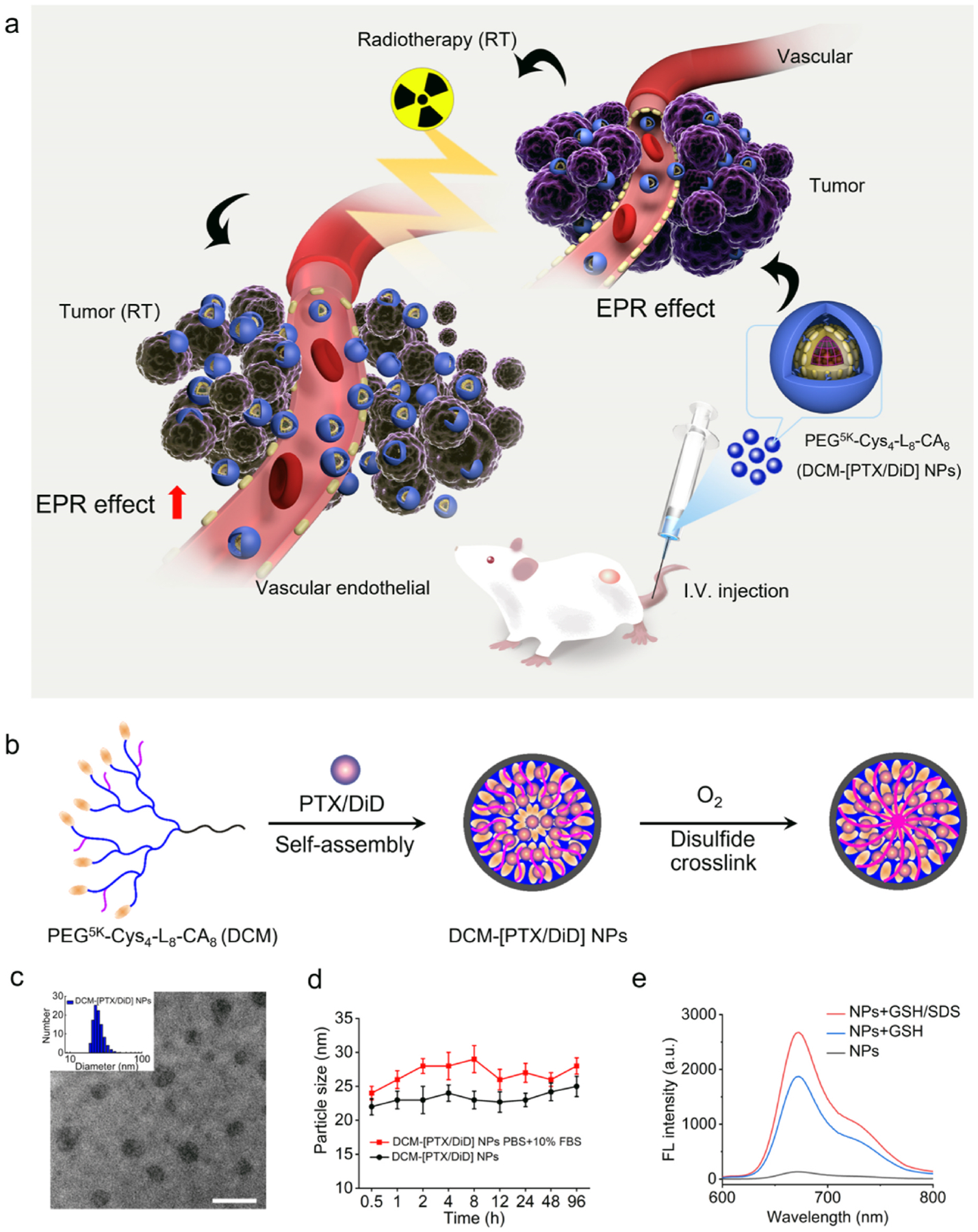
Schematic illustration and characterizations of DCM-[PTX/DiD] NPs. (**a**) Schematic illustration of self-assembled disulfide cross-linked micelles (DCMs) encapsulated PTX to improve radiotherapy in oral cancer xenograft model. (**b**) Schematic illustration of self-assembling DCM-[PTX/DiD]. (**c**) DLS size distribution and TEM images of DCM-[PTX/DiD] (stained with uranyl acetate for TEM, scale bar is 100 nm; PTX concentration: 0.1 mg/mL). (**d**) Serum stability of DCM-[PTX/DiD] in PBS solution of pH 7.4 with and without 10% FBS was measured by dynamic light scattering (incubation temperature: 37 °C; data were mean ± s.d, *n* = 3). (**e**) Fluorescence spectra of DiD dye from DCM-[PTX/DiD] in PBS, GSH and GSH + SDS conditions at 12 h (DiD concentration: 0.01 mg/mL).

Radiotherapy (RT) has been the major modality for oral cancer control, however,tumor radioresistance and normal tissue adverse effects have severelylimited the efficacy of conventional RT. Stereotactic body radiotherapy (SBRT) is a high-dose hypofractionated radiosurgery technique that precisely delivers a total dose of 20–60 Gy to solid malignant cancers in 1–5 fractions with improved tumor response. This approach was initially applied to cranial cancer in a single fraction, and recent clinical trials have mainly focused on treating thoracic cancers. SBRT is effective for local control of operable or inoperable early-stage lung cancer and significantly improves the survival rate to about 56% at 3–4 years [16,17]. At present, there are ongoing prospective clinical trials of SBRT in liver, pancreatic, prostate and kidney cancers, which have achieved good results but have not been included in clinical standard guidelines [18–20]. There have been few studies of SBRT in head and neck cancers involving recurrent and metastatic lesions [21, 22], but there is a lack of SBRT study in oral cancer. The main reason for this is that the anatomical structure of the head and neck is more complicate than that of other parts of the body, and the optimal maximum dose per fraction and the number and time interval of radiation fractions need to be further optimized [23–25]. As far as we know, there has been no systematic preclinical comparison between SBRT and conventionally fractionated radiotherapy (1.8–2.2 Gy) for head and neck cancers. In our present study, an oral cancer xenograft model was treated with DCM loaded with PTX in combination with conventionally fractionated radiotherapy (2 Gy). To monitor the biodistribution and tumor uptake of the nanoplatform *in vivo* with optical imaging, we incorporated a near infrared fluorescent hydrophobic dye DiD into DCM-[PTX] to form DCM-[PTX/DiD]. In addition, we also compare the therapeutic efficacy of DCM-[PTX/DiD] in combination with SBRT to that in combination with conventionally fractionated radiotherapy. The effect of RT in tumor uptake of DCM-[PTX/DiD] was also explored (Fig. 1a). The Superflab bolus was used to cover the tumor site to ensure an accurate dose to the irradiated subcutaneous tumor.

## 2. Results

### 2.1. Synthesis and characterization of DCM-[PTX/DiD]

The PEG^5k^-Cys_4_-L_8_-CA_8_ (PCLC) polymer was synthesized, and the average molecular weight was determined to be 11,205 (Figure S1). We prepared disulfide cross-linked micelles (DCMs) with self-assembly properties and loaded with paclitaxel (PTX) and DiD to form DCM-[PTX/DiD]. The prepared DCM-[PTX/DiD] was comprised of DCM and PTX and DiD at a 20:2:1 ratio (w/w). This nanosystem has provided a sustained release of a low dose of PTX at the tumor microenvironment (TME), which resulted in improved radiosensitivity, and the fluorescent labeling was helpful for radiotherapy planning and for determination of the tumor margins. The nanosystem was prepared as shown in the schema depicted in Fig. 1b. The DCM-[PTX/DiD] nanoparticle size (~25 nm, PDI 0.234) measured by dynamic light scattering (DLS), and the TEM image of uranyl acetate stained DCM-[PTX/DiD] in PBS are shown in Fig. 1c. The size of DCM-[PTX/DiD] in PBS (pH 7.4) with and without 10% fetal bovine serum (FBS) over 0.5–96 h at 37 °C, was measured by DLS to be rather stable (Fig. 1d). Serum stability is needed for therapeutic application *in vivo*. To assess the reversibility of the disulfide crosslink, fluorescent spectra of DCM-[PTX/DiD] in PBS with or without addition of SDS plus GSH was determined (Fig. 1e). As expected, fluorescent signal was quenched when DiD was encapsulated, but was very high when SDS and GSH were added to release from DCM-[PTX/DiD]. The PTX and DiD release profiles from DCM-[PTX/DiD] were measured by using the dialysis method. As shown in Figure S2, PTX or DiD *in vitro* release was facilitated in the presence of GSH in the intracellular level (10 mM). Release profile of PTX and DiD from DCMs were very similar; both of them were released by about 60% at 72 h.

### 2.2. Delivery and toxicity of DCM-[PTX/DiD] in vitro and in vivo

The OSC-3 human oral cancer cells were incubated with different concentrations of PTX for 8 h, and then either left untreated or treated with a single dose of 6 Gy radiation. The cells were then placed in fresh medium and further incubated for 16 h to evaluate the *in vitro* anticancer effect by MTS assay. As shown in Figure S3, PTX-loaded disulfide cross-linked micelles (DCM-[PTX]) was found to be somewhat less active than free PTX, which can be explained by the limited amount of DCM-[PTX] taken up by the tumor cells over 6 h of incubation and that not all the PTX was released and taken up intracellularly within 24 h, whereas in the case of free PTX, most of the drug was taken up by the cells within the first 6 h. However, as expected, single 6 Gy irradiation was able to shift the cytotoxic curves of both DCM-[PTX] and PTX to the left. Annexin FITC/PI flow cytometry apoptosis assay also demonstrated significant synergistic effect in inducing cell death (Figure S4). DCM-[PTX] with or without radiation resulted in 71.2% and 44.2% apoptotic cells, respectively. Compared with cells treated with DCM-[PTX] plus radiotherapy, cells treated with free PTX plus radiotherapy exhibited a slightly higher apoptosis rate.

**Fig. 2.**
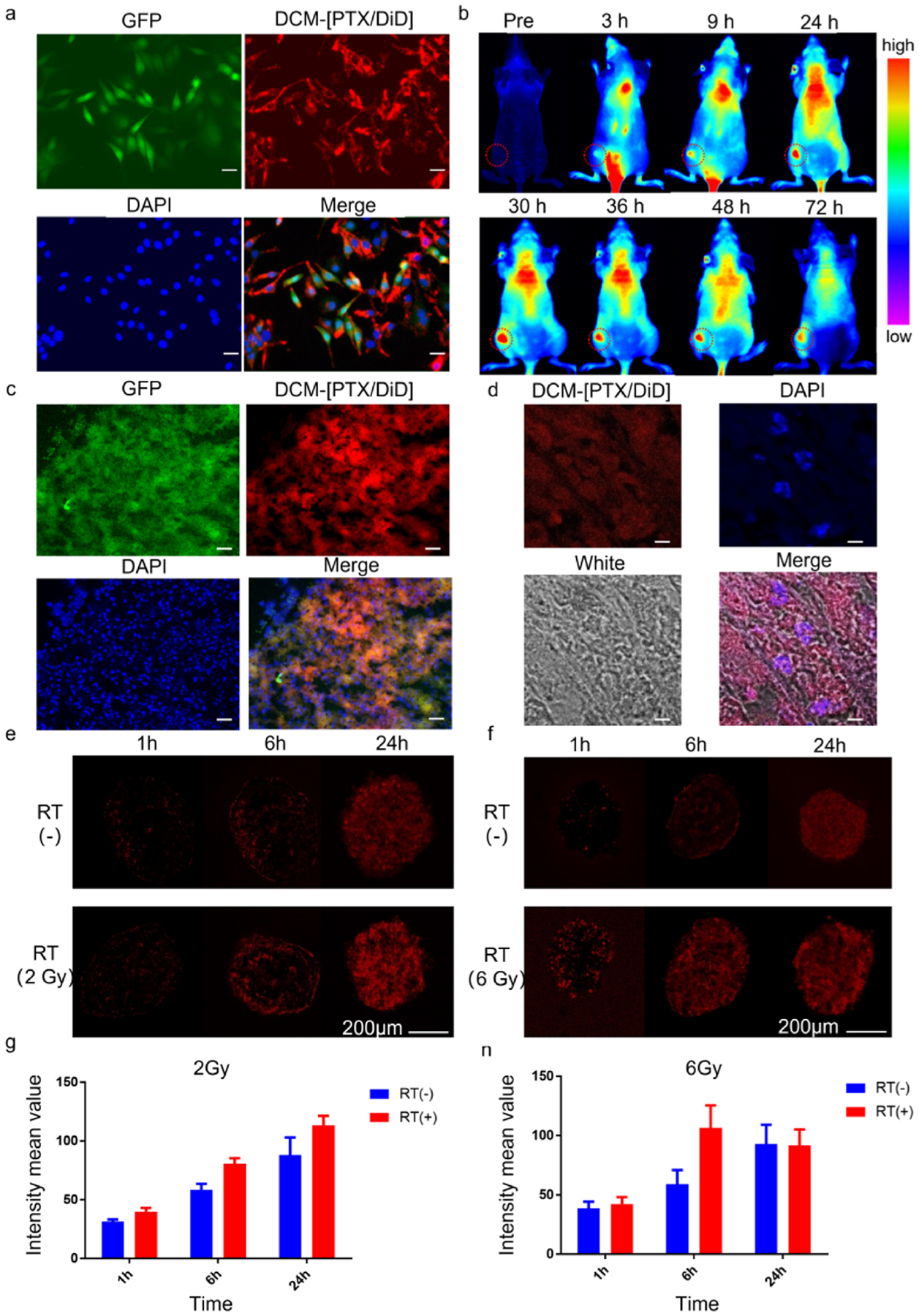
DCM-[PTX/DiD] accumulation imaging *in vitro* and *in vivo*. (**a**) OSC-3 oral cancer cellular fluorescence distribution images. (Green: OSC-3 cell line transfected with GFP; red: DCM-[PTX/DiD]; blue: DAPI; scale bar = 25 μm). (**b**) DCM-[PTX/DiD] fluorescence imaging showing tumor accumulation at different time points after tail vein injection. (**c**) Imaging of the distribution of DCM-[PTX/DiD] in the cryosection tumor tissue at 48 h after injection (green: tumor area; red: DCM-[PTX/DiD]; blue: DAPI; scale bar = 25 μm). (**d**) Uptake of DCM-[PTX/DiD] into tumor tissue under high magnification (scale bar = 75 μm). (**e-f**) Multicellular spheroids pretreated with or without radiotherapy and then incubated with DCM-[PTX/DiD] at different time points. (**e**: 2 Gy pretreated, **f**: 6 Gy pretreated, each time point *n* = 5, scale bar = 200 μm). (**g-h**) The quantitative analysis of fluorescence intensity of DCM-[PTX/DiD] in spheroids (**g**: 2 Gy pretreated, **h**: 6 Gy pre-treated).

The OSC-3 oral cancer cell line transfected with both GFP and luciferase was used to investigate the intracellular delivery of DiD-labeled DCMs by confocal laser scanning microscopy (CLSM). DiD can be considered as the surrogate drug, and was co-loaded with PTX into DCM to form DCM-[PTX/DiD]. As shown in Fig. 2a, incubation of OSC-3 cells with DCM-[PTX/DiD] for 8 h resulted in significant fluorescent uptake of DiD into the cytoplasm but not nucleus. The particle size of DCM-[PTX/DiD] was 25 nm (Fig. 1c and d), which is the optimal diameter for *in vivo* tumor penetration and accumulation. *In vivo* biodistribution of i. v. administered DCM-[PTX/DiD] was determined in the left hind leg of nude mice bearing OSC-3 xenograft, by near infra-red fluorescent imaging of the tumor sites. As shown in Fig. 2b, the fluorescent signal of the subcutaneous tumor (implanted in left flank) was first detected at 3 h after injection, with peak uptake around 30–36 h. Significant fluorescent signal remained at the tumor site even at 72 h. This result indicates that the therapeutic window available for utilizing DCM-[PTX/DiD] as a radiosensitizing agent for radiotherapy is wide, and can be exploited for clinical application. *In vivo* cellular uptake and intratumoral distribution of DCM-[PTX/DiD] was examined in a tumor excised 48 h after i. v. administration. Confocal microscopy of tumor tissue cryosections demonstrated accumulation of the red fluorescent signal of DiD at the tumor site, which correlated very well with the GFP signal of the tumor cells. (Fig. 2c & d). The tissue distribution of PTX and DiD over time after tail vein administration of DCM-[PTX/DiD] was determined. At 24, 30, 36, 48 and 72 h after injection, PTX and DiD concentrations in tumor tissue were measured by high pressure liquid chromatography-mass spectroscopy (HPLC-MS). As shown in Figure S5, accumulation concentration of PTX and DiD in the tumor tissue were higher at 30–36 h (3.15 and 1.41 μg/g tissue at 30 h; 2.53 and 1.31 μg/g tissue at 36 h), which was consistent with the fluorescence distribution intensity of DiD (Fig. 2b). Since the encapsulation of PTX and DiD was 2:1 in DCM-[PTX/DiD] NPs, and the amount of compound released at the same time point in tumor tissue was also about 2:1, it indicated that the release behavior of PTX and DiD was synchronous.

To study the effect of radiation on DCM-[PTX/DiD] accumulation in the tumor sites, we used an *in vitro* spheroid model to simulate *in vivo* solid tumor sites. OSC-3 tumor spheroids (~250 mm diameter) were first pretreated with 0 Gy, 2 Gy or 6 Gy, followed by incubation with DCM-[PTX/DiD] over different time points, and then observed under a laser scanning confocal microscope (Fig. 2e–h). The quantitative analysis of cellular accumulation of DCM-[PTX/DiD] is summarized in Fig. 2g & h. Overall, pretreatment of the spheroids with radiation was able to increase the accumulation of DCM-[PTX/DiD] inside the spheroids. This increase is particularly pronounced for cells treated with 6 Gy of radiation, with 2-fold fluorescence signal increase after 6 h. However, only a 1.3-fold increase in fluorescence signal was achieved with 2 Gy radiation after 6 h. For the spheroids treated with 6 Gy radiation, significant apoptosis was observed at 24 h, which may explain why fluorescent signal at 24 h is lower than that of 6 h. The results revealed that one 6 Gy radiation dose could enhance penetration of DCM-[PTX/DiD] inside the spheroid with increase cellular uptake as early as 1 h after radiation treatment (Fig. 2f). We also performed colony formation assay by first treated the plated cells with different radiation doses, including 0 Gy, 2 Gy, 4 Gy, 2 Gy for two times in two consecutive days (total dose 4 Gy), 2 Gy for three times in three consecutive days (total dose 6 Gy), and 6 Gy. We found that response to SBRT treatment was superior to that of the conventional fractionated radiation (Figure S6). As shown in Figure S7, assessment of the radiation induced apoptosis (green color) or necrosis (red color) were detected by CLSM.

### 2.3. Radiation improves DCM-[PTX/DiD] NPs accumulation in the tumor site in vivo

**Fig. 3.**
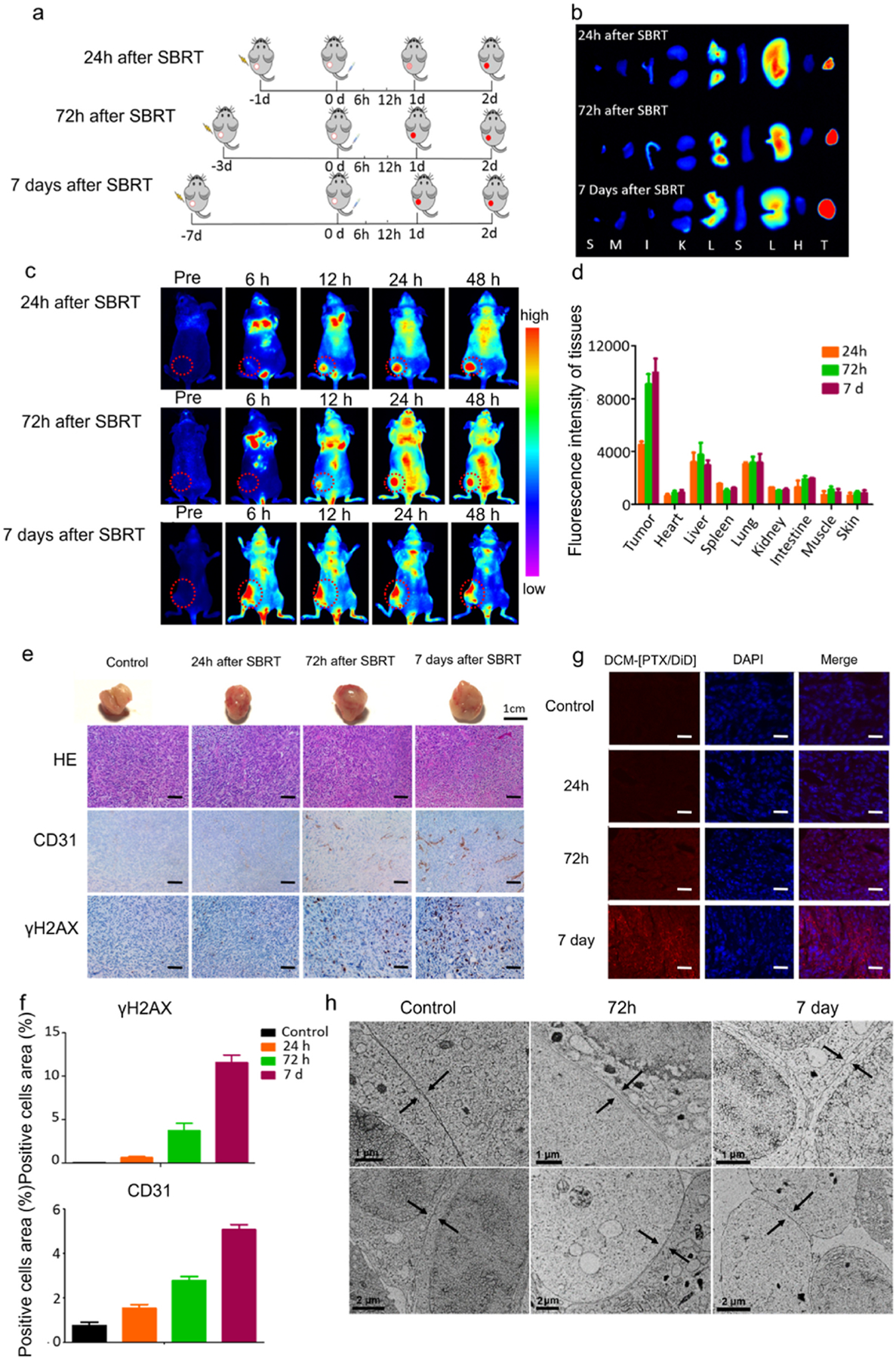
Radiation enhanced accumulation of DCM-[PTX/DiD] NPs in tumor area. (**a**) Therapeutic schema showing time interval between SBRT radiation regimen (12 Gy in single-fraction) and tail vein injection of DCM-[PTX/DiD] NPs, and subsequent *in vivo* near-infra red fluorescent imaging over 2 days. (**b**) Representative ex vivo DiD fluorescence imaging of the organs at 48 h after nanoparticle injection (T: tumor, H: heart, L: lung, S: spleen, Li: liver, K: kidney, I: intestine, M: muscle, S: skin). (**c**) Fluorescence imaging showing tumor accumulation of DCM-[PTX/DiD] in SBRT pre-treated mice at different time points after tail vein injection. DCM-[PTX/DiD] uptake was significantly higher in mice pre-treated with SBRT 72 h or 7 days earlier. (**d**) Quantitative analysis of DCM-[PTX/DiD] fluorescence signal intensity of different organs, excised 48 h after nanoparticle injection. (**e**) Gross and histological analyses of excised tumors demonstrate an increased level of tumor vascularization over time after SBRT. IHC-stained endothelial cells (anti-CD31) demonstrate the close spatial relationship of tumor vessels and NPs. γH2AX is a DNA repair marker (scale bar = 20 μm). (**f**) Quantitative immunohistochemistry analysis on the distribution of CD31 and γH2AX in tumor area by image J software. (**g**) Cryosection of tumors collected 24 h after injection of DCM-[PTX/DiD]. (red: DCM-[PTX/DiD, blue: DAPI, scale bar = 20 μm). (**h**) The border space between the tight junctions of tumor cells were detected by cryo-electron microscopy (black arrow: tight junctions between tumor cells).

To further investigate the effect of time interval after SBRT on *in vivo* uptake of DCM-[PTX/DiD] by tumors, we pretreated the tumor site with 12 Gy radiotherapy. 24 h, 72 h, or 7 d later, DCM-[PTX/DID] was administered intravenously and near infra-red fluorescent imaging of the animal performed (Fig. 3a,c). The mice pretreated with SBRT 24 h earlier showed fluorescence at tumor site peaked at approximately 48 h after nanoparticle injection, which was very similar to that of the mice without SBRT pretreatment (Fig. 2b). The mice pretreated with SBRT 72 h earlier showed the peak fluorescence intensity at approximately 24 h after nanoparticle injection. However, the mice pretreated with SBRT 7 d earlier showed that DCM-[PTX/DiD] accumulated at the tumor area within 6 h after nanoparticle injection. The results showed that changing the time interval after SBRT resulted in different degrees of DCM-[PTX/DiD] accumulation at the tumor site, which may be exploited for the development of an optimal concurrent chemoradiotherapy regimen. In these experiments, tumor tissues and major organs were excised at 48 h after injection of the NPs, and fluorescent intensity quantified under a fluorescence microscope (Fig. 3b). The 72 h-pretreated-SBRT mice and 7 d-pretreated-SBRT mice exhibited a 2-fold higher DCM-[PTX/DiD] uptake by implanted xenograft than that of the 24 h-pretreated-SBRT mice. In addition, the fluorescence signal in normal tissues showed no significant changes between groups, including the liver and kidney, which showed a relatively low fluorescence signal (Fig. 3d). We also examined the effect of SBRT on the cellular and molecular changes by histologic analysis (Fig. 3e). The gross tumor tissues and histological sections demonstrated an increased level of vascularization over time. CD31 is a marker for blood vessels. γH2AX is an important DNA double-strand break (DSB) marker for tissue exposure to ionizing radiation and it is known to have a positive correlation with the ionizing radiation dose. The quantitative analysis of CD31 and γH2AX expression is summarized in Fig. 3f, which shows strongest expression of both markers in the 7 d-pretreated-SBRT tumor. After radiotherapy, the expression of tight junction protein ZO-1 was found to decrease rapidly (Figure S8). In the contrary, the expression levels of stromal tissue markers, such as α-SMA and type I collagen, were found to increase (Figure S9). The SBRT enhanced vascular permeability and destruction of the tight junction between tumor cells in the field would allow DCM-[PTX/DiD] to readily penetrate the vascular endothelium barrier into tumor tissue. The xenograft pretreated with SBRT were collected 24 h after nanoparticle injection to further quantitatively analyze the DCM-[PTX/DiD] accumulation in the tumor area (Fig. 3g). The fluorescent signal of DCM-[PTX/DiD] (red color) was significantly increased in the 72 h and 7 d after SBRT groups compared to that of the 24 h after SBRT or the nonirradiated control groups. As shown in Fig. 3h, changes in the tight junctions were observed under cryo-electron microscopy at different time points after a single hypofractionated (12 Gy) radiation. Endothelial tight junctions are multiprotein junctional complexes that mainly prevent the leakage of transported solution. Compared to those in the control group, the space within intercellular tight junctions become wider in the 72 h-pretreated-SBRT tumor tissue, and the greatest amount of intercellular space was observed 7 d after SBRT. These ultra-structural changes would explain how DCM-[PTX/DiD] NPs were able to penetrate tumor easily a few days after SBRT. To the best of our knowledge, this is the first-time visual images of the tight junctions were obtained with cryo-EM at different time intervals after radiotherapy.

### 2.4. DCM-[PTX] greatly improved the antitumor effect of radiotherapy in nude mice bearing OSC-3 oral cancer xenografts

**Fig. 4.**
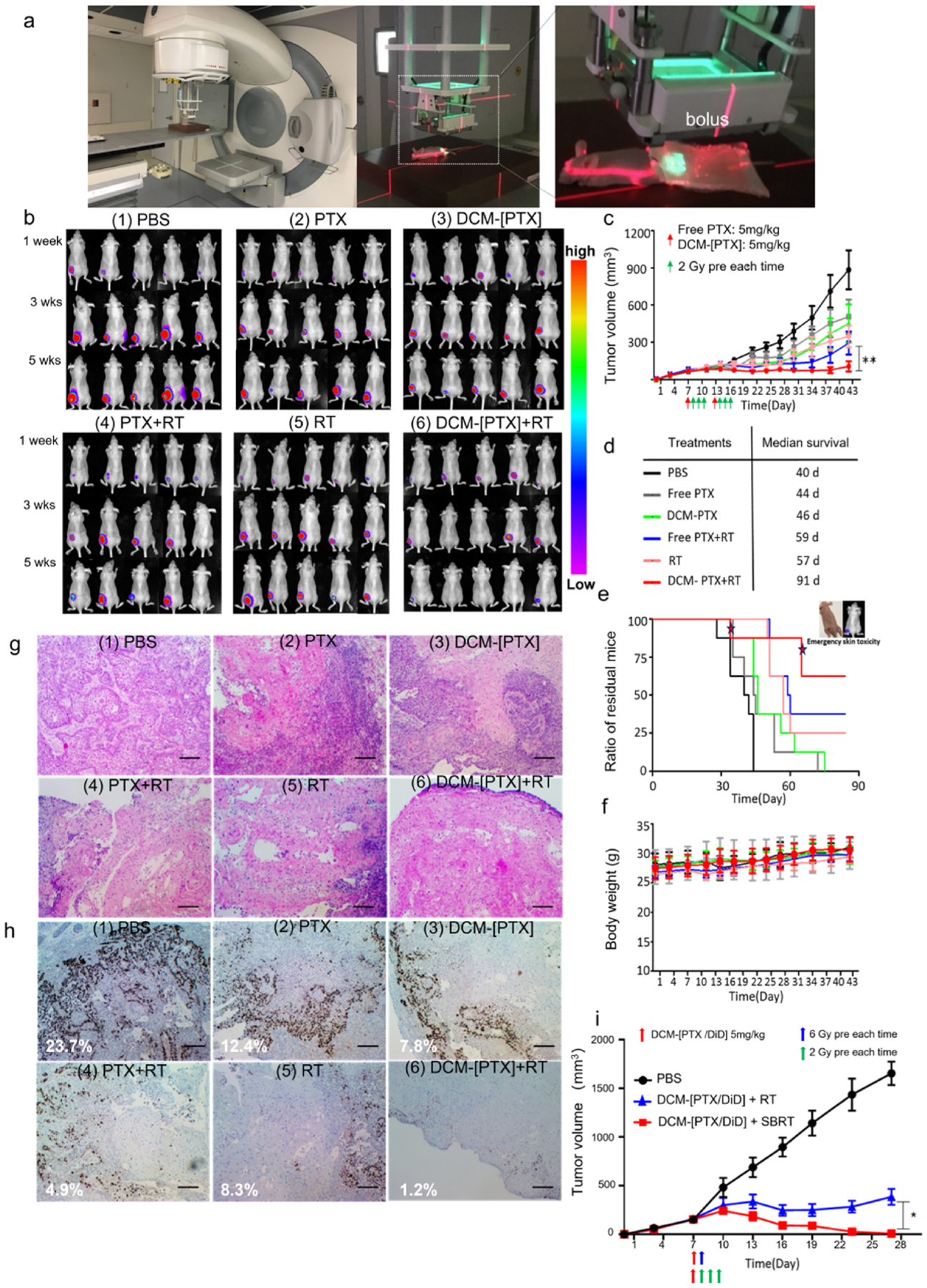
DCM-[PTX/DiD] NPs improved the anti-tumor response of radiotherapy in nude mice bearing OSC-3 oral cancer xenografts. (**a**) Mice covered with a bolus to ensure accurate dose were given 2 Gy local radiotherapy by the clinically Intensity Modulated Radiation Therapy (IMRT) Elekta linear radiotherapy accelerator (Dose of PTX: 5 mg/kg; radiation dose: 2 Gy). (**b**) Bioluminescence monitoring of nude mice bearing OSC-3 oral cancer xenograft (*n* = 8). Images of five mice/group are shown in weeks 1, 3, and 5. Nude mice were inoculated with OSC-3 cells transfected luciferase gene, randomly divided into six groups: (1) PBS, (2) PTX, (3) DCM-[PTX], (4) RT + PTX, (5) RT, (6) RT + DCM-[PTX]. (**c**) Tumor growth curves of mice (*n* = 8) bearing OSC-3 oral tumors. Red arrow depicts injected 5 mg/kg PTX/DCM-[PTX] and green arrow depicts 2Gy local radiotherapy (total dose: 12 Gy). Differences between RT + PTX and RT + DCM-[PTX] were evaluated by the *t*-test (**p < 0.01). (**d-e**) Mice median survival time point were listed in the table. (Two stars in residual mice curve represented mice excluded because of emergency skin toxicity). (**f**) Changes of the nude mice body weight. (**g-h**) H&E histological and Ki67 immunohistochemistry staining evaluation of tumor nodules after treatment, demonstrating chem/radiotherapy effectively inhibit tumor proliferation and induce apoptosis (scale bar = 20 μm). (**i**) Tumor growth curves of nude mice bearing OSC-3 oral tumors treated with DCM-[PTX/DiD] in combination with SBRT or conventional radiation. (*n* = 6, Red arrow represents tail intravenous injection 5 mg/kg DCM-[PTX/DiD], blue arrow and green arrow means 6 Gy and 2 Gy local radiotherapy, respectively). Data are presented as mean ± s.d.*p < 0.05.

Encouraged by the synergistic antitumor response *in vitro* and the optimal biodistribution of DCM-[PTX/DiD] *in vivo*, we further explored the wide window of therapeutic efficacy *in vivo*. In our experiment, the mice were randomly divided into six groups (*n* = 8 per group): (1) PBS (untreated), (2) PTX alone, (3) DCM-[PTX] alone, (4) radiation (RT) alone, (5) combination PTX with radiation, and (6) combination DCM-[PTX] with radiation. When the tumor volume reached 100 mm^3^, low-dose DCM-[PTX] (PTX equivalent: 5 mg/kg) was administered intravenously. 24 h later, the tumor area was locally irradiated with 2 Gy of radiation (6 MV, Elekta) for three consecutive days with a clinical intensity modulated radiation therapy (IMRT) linear radiotherapy accelerator. The same treatment cycle was repeated in the second week. To be fully effective, radiosensitizer needs to be at the tumor sites during radiotherapy. The proven prolonged retention of DCM-[PTX] at the tumor site for longer than 72 h (Fig. 2b) makes it possible that such radiosensitizer can be given once a week followed by daily radiation. For the clinical translational research, the Elekta external beam radiotherapy device of the Radiation Oncology Department of the Medical Center was used in our study. As shown in Fig. 4a, the cross hairs of the red laser were adjusted to target the center of the tumor site, and the green area shows the irradiated field. The irradiated tumor region was covered by a bolus, which is a waxy tissue material placed on the tumor surface to ensure the accuracy of the ionizing radiation dose. The fractionated dose used in our study was similar to the NCCN guidelines for clinical cancer radiotherapy, and nude mice bearing oral cancer xenografts were irradiated 6 times with 2 Gy each time.

Oral cancer xenografts were used to validate the therapeutic responses to PBS, PTX, DCM-[PTX], RT, PTX + RT, and DCM-[PTX]+RT (*n* = 8). Treatment schedule is shown in Fig. 4c, and low dose PTX at 5 mg/kg was used as radiosensitizing agent in this experiment. Treatment efficacy was measured by the change in the tumor volume (Fig. 4c), and the median residual tumor burden is summarized in Fig. 4 d–e. All treatment groups showed obvious tumor growth delay after day 16 in comparison with the control group. Radiation alone, PTX alone, and DCM-[PTX] alone groups all showed similar modest therapeutic response. Combination radiation with free PTX or with DCM-[PTX] both showed significant inhibition in tumor growth. By day 42, most tumors remained suppressed in the combination radiation with DCM-[PTX] group. Bioluminescence images were used to dynamically monitor the changes in tumor burden in different treatment groups (Fig. 4b). Images of five mice per group are shown for week 1, 3, and 5. Representative photographs of the tumor volume in the (RT + PTX) group and (RT + DCM-[PTX]) group at 7 weeks are shown in Figure S10. The blue arrow indicates that the tumor was completely eliminated, and the red arrow indicates that residual small tumor still existed after treatment. Based on our statistical analysis, five mice in (RT + DCM-[PTX]) group were cured, including four mice cured within 7 weeks and one cured in 8 weeks. However, only three mice were cured in the (RT + PTX) group. Compared with the (RT + PTX) group, the (RT + DCM-[PTX]) group showed an approximately 1.7-fold improvement in efficacy. The analysis of the ratio of residual tumor in mice is shown in Fig. 4d–e, with the median survival indicated in the table. For the survival curve in Fig. 4e, the two asterisks in the (RT + DCM-[PTX]) group indicates that two mice were excluded from analysis because they were euthanized due to radiotherapy-induced skin ulcers. As shown by the representative groups in Figure S10, the tumors in the two mice with skin toxicity also significantly shrank in (RT + DCM-[PTX]) group and might even have disappeared. One of the five cured mice in (RT + DCM-[PTX]) group showed tumor recurrence on 91 d, and the median residual tumor burden of this group was significantly lower than that of the other treatment groups. Evaluation of the systemic toxicity in terms of body weight loss revealed that there was no obvious toxicity in any of the treatment groups (Fig. 4f). As shown in Figure S11, blood was drawn after two cycles of treatment to test the blood chemistry and liver/kidney function. The results revealed that all blood indicators were almost within the normal range.

After two cycles of treatment, the tumor tissues were collected for H&E and immunohistochemical staining. The synergistic anticancer efficacy of DCM-[PTX] and radiation further confirmed the destruction of tumor cells at the tumor site (Fig. 4g). As shown in Fig. 4h, all treatment groups suppressed tumor proliferation in comparison with the control group. There were 1.24% positive Ki67+ areas in the (RT + DCM-[PTX]) group compared with 4.8% positive Ki67+ areas in the (PTX + RT) group. The results indicate that RT + DCM-[PTX] could better inhibit cell proliferation.

Based on the excellent anti-tumor efficacy shown in our previous treatment trials, we further investigate the combined effect of hypofractionated radiotherapy and DCM-[PTX/DiD]. When the tumor volume reached 150 mm^3^, the mice were treated with DCM-[PTX/DiD], followed the next day by hypofraction radiotherapy (SBRT, one session of radiation with 6 Gy) or conventional fraction radiotherapy (2 Gy each day for 3 consecutive days). As shown in Fig. 4i, SBRT in combination with DCM-[PTX/DiD] was more efficacious than conventional radiotherapy with DCM-[PTX/DiD], with more rapid and complete response. Importantly, systemic toxicity was not observed in either of the treatment arms (Figure S12a). After one cycle of combination treatment, tumors were excised for H&E and Ki67 staining, which shows minimal residual Ki67+ cells in the combination SBRT and DCM-[PTX/DiD] arm (Figure S12b). To further investigate the necrosis and mitotic catastrophe, Caspase3 and Mad2 were stained, respectively (Figure S13). For *in vivo* experiment, high dose radiation was prone to induce necrosis. The mitotic catastrophe marker Mad2 was found to correlate negatively with radiation dose.

## 3. Discussion

We investigated disulfide cross-linked micelle (DCM) encapsulating PTX and DiD fluorescent dye in combination with conventional fraction radiotherapy (2 Gy per fraction) or SBRT (6 Gy per fraction) in an oral cancer xenograft model. Despite improvements in the diagnosis and surgical methods widely applied in oral cancer and other head and neck cancers, effective treatment however remains a challenge. There are complex anatomical structures surrounding sites of oral cancer, which makes it difficult to completely resect the tumor tissue due to vascular and perineural invasion, positive surgical margins, and lymphovascular invasion [26–28]. Postoperative oral cancer patients with high-risk pathological features will develop local recurrence, and remote metastasis, leading to a 5-year survival rate of only 50% [29]. About 50% of the patients who underwent surgical resection will require radiation therapy [30]. Radiation therapy with a total dose ranging from 50 Gy (1.6–2.0 Gy per fraction) to 70 Gy (2.0–2.2 Gy per fraction) has been considered as a standard treatment for oral cancer according to the National Comprehensive Cancer Network (NCCN) guidelines. Concurrent radiotherapy and chemotherapy are more efficacious but much more toxic than conventional fraction radiotherapy alone in advanced head and neck cancer patients [31]. The disulfide crosslinked nanotaxane reported here allows preferential delivery of PTX to tumor sites via EPR effects and prolonged retention of the NPs at the tumor sites but not normal tissues, making it an excellent chemotherapeutic formulation for combination chemo-radiation therapy.

For many of the preclinical studies reported here, we included DiD into our DCM-[PTX] nanoformulation to form DCM-[PTX/DiD] so that the tissue distribution of DiD, the surrogate drug, can be monitored by optical imaging and fluorescent microscopy during biodistribution and therapeutic studies. DCM-[PTX/DiD] has several desirable attributes that align with clinical applications. For example, the ~30 nm size range of DCM-[PTX/DiD] favors *in vivo* tumor uptake via EPR effects. The intramicellar disulfide crosslinking improves the *in vivo* stability of the NPs, minimizes premature drug release during blood circulation, and prolongs the retention of the NPs at the tumor site ranged from 3 d to 5 d. Our DCM have a high PTX-loading capacity and can achieve almost 100% PTX loading at 5 mg/mL [32], while ensuring that the NPs are at the optimal particle size (~30 nm). The disulfide cross-linked micelles (DCM-[PTX/DiD]) dissociate when delivered to the tumor sites or inside the tumor cells with high GSH concentrations [32,33]. Compared with PTX released from Taxol, PTX was released from DCMs at a much slower rate [7]. NK105, a non-crosslinked 85 nm micellar nanoformulation of PTX prepared by self-assembling of polyethylene glycol (PEG)-polyaspartate polymer, had been used at a dosage of 45 mg/kg to improve radiosensitivity [34,35]. Our ~25 nm DCM-[PTX/DiD] were found to be highly efficacious as a radiosensitizer at a PTX dosing of only 5 mg/kg. The strong near infrared fluorescence signal of DCM-[PTX/DiD] at the tumor sites was found to be detectable 3–5days after intravenous injection. With appropriate longer wavelength hydrophobic dyes, the nanotaxane can potentially be used for image-guided surgery, and image-guided radiotherapy of oral cancer [36,37]. We have previously demonstrated that cancer targeting ligands can improve therapeutic efficacy of DCM-[PTX] in ovarian cancer xenograft model [38]. This can certainly be applied to combination chem-radiotherapy.

It is well known that combination chemo-radiation (e.g. combination Taxol with radiation) is toxic and difficult for the patient to tolerate [39–41]. It is gratifying to see that the mice treated with combination DCM-[PTX/DiD] (5 mg/kg of PTX) and radiotherapy did not lose any weight compared with the control group (Fig. 4f). Blood samples were obtained from mice after two cycles of concurrent chemotherapy and radiotherapy to further examine the systemic toxicity, by assessing red blood cells (RBCs), white blood cells (WBCs), platelets (PLTs), hemoglobin (HGB), as well as liver and kidney function tests, which showed no significant abnormalities. This lack of systemic toxicity is consistent with our previous therapeutic studies with DCM-[PTX] given at 30 mg/kg of PTX, 6 times the dose used in this current chemo-radiation study [32,42,43]. A 0.5 cm-thick bolus was utilized in our subcutaneous xenograft model to ensure accurate radiation dose on the surface. In clinical practice, bolus is commonly used for radiotherapy of superficial tumors, such as those in patients with radical mastectomy or tumors invading the skin. For the combination chemo-radiation therapeutic efficacy study shown in Fig. 4b–h, the mice were treated with free PTX or DCM-[PTX/DiD] followed by three consecutives daily 2 Gy radiation. The treatment cycle was repeated a week later for a total of two cycles. As expected, the therapeutic response was found to be much better for the combination DCM-[PTX/DiD] and radiotherapy arm. The prolonged retention of DCM-[PTX/DiD] at the tumor sites had allowed radio-sensitization to occur beyond the day of PTX administration. SBRT has been used clinically for post-operative radiation therapy of head and neck cancer with a total dose of 36 Gy in six fractions over two weeks, i. e. 6 Gy per fraction [44]. Here, we compared the radiosensitization effect of DCM-[PTX/DiD] (5 mg/kg) on radiotherapy given at conventional dose (2 Gy × 3 fractions) vs SBRT (6 Gy × 1 fraction). The latter regimen was found to be more efficacious (Fig. 4i). Clinical SBRT has several advantages over conventional radiotherapy for oral cancer therapy. First, hypofractionated radiotherapy will decrease the frequency of radiotherapy, thereby reducing the possibility of positioning error during the treatment. Second, SBRT as an optimal treatment is likely to help patients shorten the radiotherapy time, improve the efficiency of radiotherapy and relieve the economic burden to the patients. Third, oral cancer is relatively distant from the important dose-limiting structures (brain and eye lens) in the head, and high-dose irradiation is more conducive to tumor regression. Although SBRT may induce severe acute side effects, the current phase II GORTEC 2017–03 trial study has reported that the safety of postoperative SBRT in early-stage oral cancer is similar to that of conventional radiotherapy [45]. DCM-[PTX/DiD] combined with SBRT is expected to further reduce systemic toxicity and achieve much better radioenhancement. Fourth, SBRT has been shown to enhance the magnitude of the immune response by producing more neoantigens, which could recruit a large number of T cells and promoted the release of inflammatory mediators related to tumor apoptosis, such as IFNγ, TNF-α, TGβ−1 and interleukins. Many immunotherapies combined with SBRT can improve the treatment response, including immune checkpoint blockade, small-molecule activators, peptides and vaccines [46–48]. Mini-beam radiation as the third-generation synchrotron apparatus has a smaller beam size, effectively improving the useful diffraction and the focus beam with the high damage ratio could reduce the side effect of normal tissues. Combined with mini-beam radiation, William Zamboni’s recent work produced a 7.1-fold greater increase in the tumor delivery of NPs in triple-negative breast cancer xenograft model, which could further enhance the synergistic effect of chemo-radiotherapy [49].

The synergistic anti-cancer effects between ionizing radiation and PTX is well-established. Here we have discovered that ionizing radiation can also increase the uptake of DCM-[PTX/DiD] NPs into the tumor sites. As shown in Fig. 2e–f, the fluorescence images of cellular spheroids demonstrated that DiD-labeled DCM NPs were delivered into cells and accumulated 1.2 to 2-fold after a single ionizing radiation dose. Compared to conventional irradiation at 2 Gy, 6 Gy of irradiation significantly increased DCMs uptake in cellular spheroids at 6 h. Therefore, the results of the *in vitro* experiment made us realize that the different doses of Refionizing radiation and intervals of radiotherapy may affect the accumulation of DCMs in the tumor site, thereby affecting the efficacy of DCM-[PTX/DiD] as a radiosensitizer. To the best of our knowledge, this is the first study to report that fluorescent images showed the NPs biodistribution after SBRT at different time intervals. The 72 h-pretreated SBRT and 7 d-pretreated SBRT tumor tissues showed that DCM-[PTX/DiD] was taken up at more than twice the amount compared to that of the 24 h-pretreated SBRT tumor. The improved intra-tumoral distribution of DCM-[PTX/DiD] after radiation treatment may result from the increased EPR effect, destruction of vascular endothelial cells and enhanced intercellular diffusion [50]. However, in our study, the gross tissue and histological samples demonstrated that the expression of vascular epithelial protein was higher and blood vessels were more abundant in the treated groups compared to those in the control group. The intercellular changes in tight junctions were detected by cryo-electron microscopy, as shown in Fig. 3h. Apoptotic cells consume less oxygen and show increased intercellular space, which provides more oxygen to surviving cells. In addition, a study reported that paclitaxel not only arrested the cycle at G2/M but also effectively reduced tissue interstitial fluid pressure. Ionizing radiation has been shown to alter tumor microenvironment by interfering cell-cell communication and inducing stromal cells to secrete various cytokines, ɑ-SMA, MMP-2 and MMP-9, which can potentially promote the metastasis of cancer [51,52]. On the other hand, radiation can destroy the tight junctions between tumor cells, widen the intercellular space and improves the penetration of NPs into the tumor [53]. Although the underlying biological mechanism of radiation-induced tight junction reduction is unclear, radiotherapy with concurrent chemotherapy has been shown in many clinical studies to consistently prolong the survival rate compared chemotherapy alone [54–56].

The oral cancer preclinical results demonstrated that disulfide cross-linked micelles encapsulating PTX and DiD in combination with radiation treatment showed improved therapeutic effects and therefore warrant clinical development. The demonstrated therapeutic advantage of combination SBRT with DCM-[PTX/DiD], the prolonged retention of nanotaxane at the tumor sites, and the dynamic effects of ionizing radiation on facilitating *in vivo* uptake of nanotaxane into the tumor will be exploited for rational design of protocol for clinical development.

## 4. Materials and methods

### 4.1. Materials

Monomethylterminated poly (ethylene glycol) monoamine (MeOPEG-NH_2_, Mw: 5000 Da) was purchased from Rapp Polymere (Germany). Paclitaxel (Mayne Pharma, NJ, USA) was obtained from UC Davis Comprehensive Cancer Center. 1,10-dioctadecyl-3,3,30,30-tetramethylindodicarbocyanine perchlorate (DiD, red, Product No. D7757) and 4, 6-diamidino-2-phenylindole (DAPI, blue, Product No. D1306) was obtain from Invitrogen. MTT [3-(4,5-dimethyldiazol-2-yl)-2,5 diphenyl tetrazolium bromide] (Product No. CT02) and other chemicals were obtained from Sigma-Aldrich (St. Louis).

#### 4.1.1. Synthesis telodendrimers and self-assembly preparation of NPs

The thiolated telodendrimer (named as PEG^5k^-Cys_4_-L_8_-CA_8_, abbreviated as PCLC) was synthesized according to our previously report [5]. Briefly, a third generation of dendritic polylysine terminated with four Boc and Fmoc groups on one end of PEG firstly was synthesized f. After the removal of Boc groups with 50% (v/v) trifluoroacetic acid (TFA) in dichloromethane (DCM), (Fmoc)Cys (Trt)-OH, (Fmoc)Ebes-OH and Cholic acid NHS esterwere coupled step by step to the terminal end of dendritic polylysine. The Trt groups on cysteines were removed by TFA/H_2_O/ethanedithiol (EDT)/triethylsilane (TIS) (94:2.5:2.5:1, v/v) resulting in PCLC thiolated telodendrimer. The thiolated telodendrimer was recovered from the mixture by three cycles of dissolution/reprecipitation with DMF and ether, respectively. Finally, the thiolated telodendrimer was dissolved in acetonitrile/water and lyophilized.

#### 4.1.2. Preparation of PTX and DiD loaded NPs

Hydrophobic PTX and/or DiD were loaded into NPs by the solvent evaporation method. 20 mg PCLC telodendrimer along with different amounts of PTX and/or DiD were first dissolved in CHCl_3_/MeOH, mixed and evaporated on rotavapor to obtain a homogeneous dry polymer film. The film was reconstituted in 1 mL PBS, followed by sonication for 30 min, allowing the sample film to disperse into NPs solution. Finally, the PTX or DiD-loaded NPs solution was filtered with a 0.22-μm (Sigma, USA) filter to sterilize the sample.

#### 4.1.3. The characterization of NPs

The size and size distribution of DCM-[PTX/DiD] NPs were measured by dynamic light scattering instruments (DLS, Microtrac, USA). The micelles concentration was at 1.0 mg/mL and PTX concentration at a dose of 0.1 mg/mL. The morphology images of NPs were measured under CM-120 transmission electron microscope (TEM, Philips, Holland). The data of NPs was presented as mean + standard deviation. The stability study of DCMs loaded DiD was monitored the change of particle size with or without sodium dodecyl sulfate (SDS) and GSH, which has been reported effectively induced the disintegration of polymeric micelles [57]. The particle size of PTX-loaded DCMs was incubated with PBS solution with or without 10% FBS at body temperature (37 °C) by DLS.

### 4.2. In vitro cellular experiments

Human oral cancer cell line OSC-3 established from metastatic lymph nodes was gifted from department of orofacial sciences in the University of California San Francisco to use in both *in vitro* and *in vivo* studies [58]. OSC-3 cells were grown in DMEM (Product No. 11965084, Gibco) containing 10% FBS and 1% penicillin (Product No. 10378016, Gibco) at 37 °C in humidified cell incubator (Thermo Fisher, USA) with 5% CO_2_. To assess cellular toxicity, cells were seeded at a density of 1 × 10^4^/well in 96-well plates (*n* = 3, Biosciences, MA, USA) for 24 h. Then, PTX or DCM-[PTX] (PTX concentration 0.1 mg/mL) with different dilution were co-cultured with cells for 8 h, followed by irradiation with 6Gy (X-ray cabinet, USA). After further incubation for 16 h, cells were washed with PBS 3 times and MTT reagent was then added into each well to evaluate the relative cell viability under a Micro-plate ELISA reader (SpectraMax M2, USA). Cell viability was calculated as follow: (OD_490nm_ of treatment/OD_490nm_ of blank control) X100% = cell viability (%). To demonstrate cellular apoptosis, cells were treated with PTX or DCM-[PTX] in 6-well plates for 8 h and followed by irradiation with 6 Gy. Cells were then stained with PI and Annexin V for 30min to monitor apoptosis by LSR Fortessa flow cytometry (Biosciences, MA, USA). The flow cytometry data was analyzed by FlowJo software (Ashland, USA) and the gating strategy depend on SSC and FSC properties. To assess cell uptake, OCS-3 oral cancer transfected with GFP (green fluorescence, Sigma-Aldrich, USA) were incubated with DCM-[PTX/DiD] (red fluorescence) in 8-well chamber slides (Biosciences, MA, USA) for 8 h, after which the cells were washed with PBS 3 times and then stained with DAPI for half an hour. DCM-[PTX/DiD] NPs delivered inside the cells was detected by the fluorescent microscope. An Apoptosis/Necrosis Detection kit (ab176749) was utilized to evaluate the apoptosis and necrosis at different radiation doses. Cells were exposed to 0 Gy, 2 Gy, 4 Gy, 6 Gy, 8 Gy, and further incubate with fresh medium for 24 h. After incubate with the kit ingredients for 10 mins, healthy tumor cells (blue color), apoptotic tumor cells (green color) and necrotic cells (red color) were detected by CLSM.

#### 4.2.1. In vitro cell spheroids penetration

OSC-3 cells were seeded in specific U-shaped bottom 96-well plate (Biosciences, MA, USA) at a density of 2 × 10^4^. There developed only one spheroid in each well. The formed spheroids were pre-irradiated with 2 Gy or 6 Gy, and incubated with DCM-[DiD] for 24 h. The cell pellets (*n* = 5) were then examined for fluorescence by confocal microscopy. For each spheroid, images of 4 different layers along the z-axis was obtained and their fluorescence value averaged. This has allowed us to assess the penetration ability of nanomaterials into the spheroids.

#### 4.2.2. In vitro cell colony formation assay

A total of 5 × 10^2^ OSC-3 cells were seeded in 35 mm dishes at room temperature. After 24 h incubation, the cells were treated with different radiation dose, as following: 0 Gy, 2 Gy, 4 Gy, 2 Gy for two times in two consecutive days (total dose 4 Gy), 2 Gy for three times in three consecutive days (total dose 6 Gy), 6 Gy, which have beneficial to evaluate treatment response between SBRT and conventional radiotherapy. The medium was exchanged after radiation and further replaced every 3 days. After 10 days incubation, the colonies were appeared in dishes and were washed with PBS for 2 times. Then, the colonies fixed with methanol for 20 mins and stained with 0.5% crystal violet for 30 mins. The number of colons (defined as more than 50 cells) were counted manually. The independent experiments were repeated for 3 times.

### 4.3. Animal model

All mouse experiments were approved by the Institutional Animal Use and Care Committee of the University of California Davis (IACUC) and in accordance with NO. 15315. Female nude mice with 6–8 weeks of age were purchased from Charles River Laboratories (CA, USA) at room temperature with 50–70% humidity. The left hind leg of nude mice were implanted subcutaneously with OSC-3 oral cancer cells (5 × 10^6^) suspended in 50 μL mixture volume of PBS and Matrigel (Cat.No. A1413201, Thermo Fisher, USA).

#### 4.3.1. In vivo/ex vivo fluorescence imaging

Female nude mice with subcutaneous oral cancer xenograft were given intravenously DCM-[PTX/DiD] NPs (DiD concentration: 1.25 mg/mL) to detect the *in vivo* distribution. The post-injection anesthetic mice were recorded the change of fluorescence signal at different time points under Kodak imaging system (IS2000MM, USA) with excitation at 625 nm. After 24 h or 48 h post-injection, the major organs and tumors were collected and the fluorescence intensity quantified.

#### 4.3.2. In vivo treatment study

When the subcutaneous tumor volume reached almost 100 mm^3^, nude mice bearing OSC-3 oral cancer were randomly divided into six groups (*n* = 8): control (PBS), free drug (PTX), DCM-[PTX], radiotherapy (RT, 2Gy), RT + PTX, RT + DCM-[PTX]. The dose of PTX was 5 mg/kg, DCM NPs was 50 mg/kg (the concentration based on polymer). 24 h after tail-vein injection, the tumors were irradiated at 2 Gy for three times by linear radiotherapy accelerator (6 MV, Elekta). The irradiated tumor field was covered with the superflab bolus (Radiation products design, Inc, USA) to increase the targeted radiation dose. Mice with oral cancer xenograft were treat once every week for two weeks. In order to further compare the treatment efficacy of conventional radiotherapy and SBRT, we designed the secondary experiment when tumor volume reached 150 mm^3^. Mice were treated with PBS, DCM-[PTX] combined with 2 Gy radiotherapy for three times, and DCM-[PTX] combined with one 6 Gy hypofraction, respectively. The body weights and tumor volume were recorded twice a week and calculated as follows: Volume (mm^3^) = 0.5 * length (mm) * width^2^ (mm^2^). During the irradiation period, mice were injected intraperitoneal with luciferin (Sigma-Aldrich, USA) to dynamically monitor the changes of bioluminescent signal of tumor site.

#### 4.3.3. In vivo toxicity of therapeutic toxicity

To evaluate for the toxicity of chemoradiotherapy, blood was draw from nude mice bearing oral cancer xenograft after 2 cycles of treatment (PTX 5 mg/kg). The blood samples were sent to the Comparative Pathology Laboratory of University California, Davis for complete blood count (CBC) and liver/kidney function.

#### 4.3.4. H&E and IHC evaluation

Mice bearing oral cancer xenograft were sacrificed after two cycles of treatment and all treated tumors were collected to investigate therapeutic effects. The samples were stained with Haematoxylin and eosin (H&E). Tumor tissues were processed for immunohistochemistry by the following antibodies: rat monoclonal to CD31 (eBioscience, antibody dilution 1:400) and mouse monoclonal to γH2AX (Millipore, antibody dilution 1:300). The Decloaking Chamber (Biocare Medical, Concord, CA) with citrate buffer was used to perform antigen retrieval. The tumor samples were incubated with primary antibody at room temperature overnight in the humidified cassette. After goat serum blocking, the secondary antibody (Vector Labs, Burlingame, CA, antibody dilution 1:1000) was used with ABC Kit and DAB Kit (Vectors Lab). The positive area in the field was measure by image J software.

### 4.4. Statistical analysis

Student’s t-test for two groups was compared between two groups. One-way ANOVA was analyzed for multiple-group. The data was presented as mean +/− standard deviation. Statistical significance was considered as *P < *0.05*, **P < *0.01*, ***P < *0.001*.

## Supplementary Material

1

## Data Availability

Data will be made available on request.
