# Supplementary material for: Nanoradiosensitizer with good tissue penetration and enhances oral cancer radiotherapeutic effect
Source: Biomaterials. Author manuscript; Available in PMC 2023 Mar 17. (PMC10021813; doi:10.1016/j.biomaterials.2022.121769)
Supplement: 1 [file NIHMS1879286-supplement-1.docx]

Supporting Information

Nanoradiosensitizer with good tissue penetration and enhances oral cancer radiotherapeutic effect

Di Jing^1,2^, Nian Jiang^5^, Fengyi Wang^3^, Chunping Mao^3^, Shujun Han^4^, [Pui Yan Ho](https://pubmed.ncbi.nlm.nih.gov/?term=Ho+PY&cauthor_id=31694904)^1^, Wenwu Xiao^1^, Yuanpei Li^1^, Jian-Jian Li^4^, Lu Zhang^3,^*, Kit S Lam^1,6^***

1. Department of Biochemistry and Molecular Medicine, UC Davis Comprehensive Cancer Center, University of California Davis, Sacramento, California, USA.
2. Department of Oncology, National Clinical Research Center for Geriatric Disorders, Xiangya Hospital, Central South University, Changsha, Hunan, China.
3. Department of Biomedical Engineering, Southern University of Science and Technology, Shenzhen, Guangdong, China.
4. Department of Radiation Oncology, School of Medicine, University of California Davis, Sacramento, California, USA.
5. Department of Neurosurgery, Xiangya Hospital, Central South University, Changsha, China.
6. Division of Hematology and Oncology, Department of Internal Medicine, School of Medicine, University of California Davis, Sacramento, California, USA.

Email: kslam@ucdavis.edu; zhanglu@sustech.edu.cn.

Keywords: radio-sensitizer, nano-micelle, radiotherapy, oral cancer

**
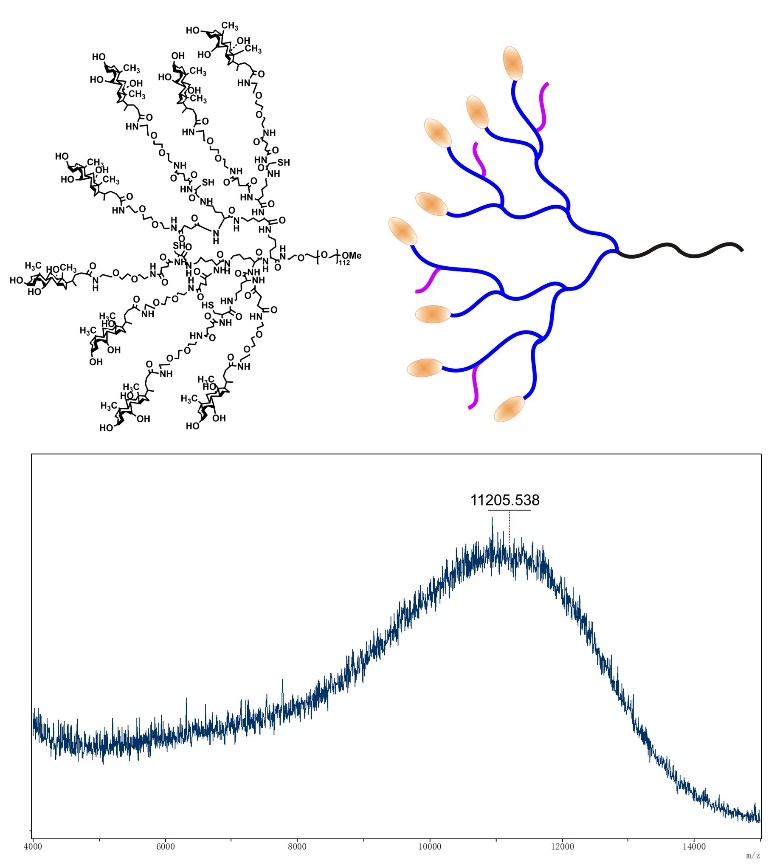
**

**Figure S1.** Chemical characterization of PCLC telodendrimer: Chemical structure, schematic illustration, mass spectra *via* MALDI-TOF.


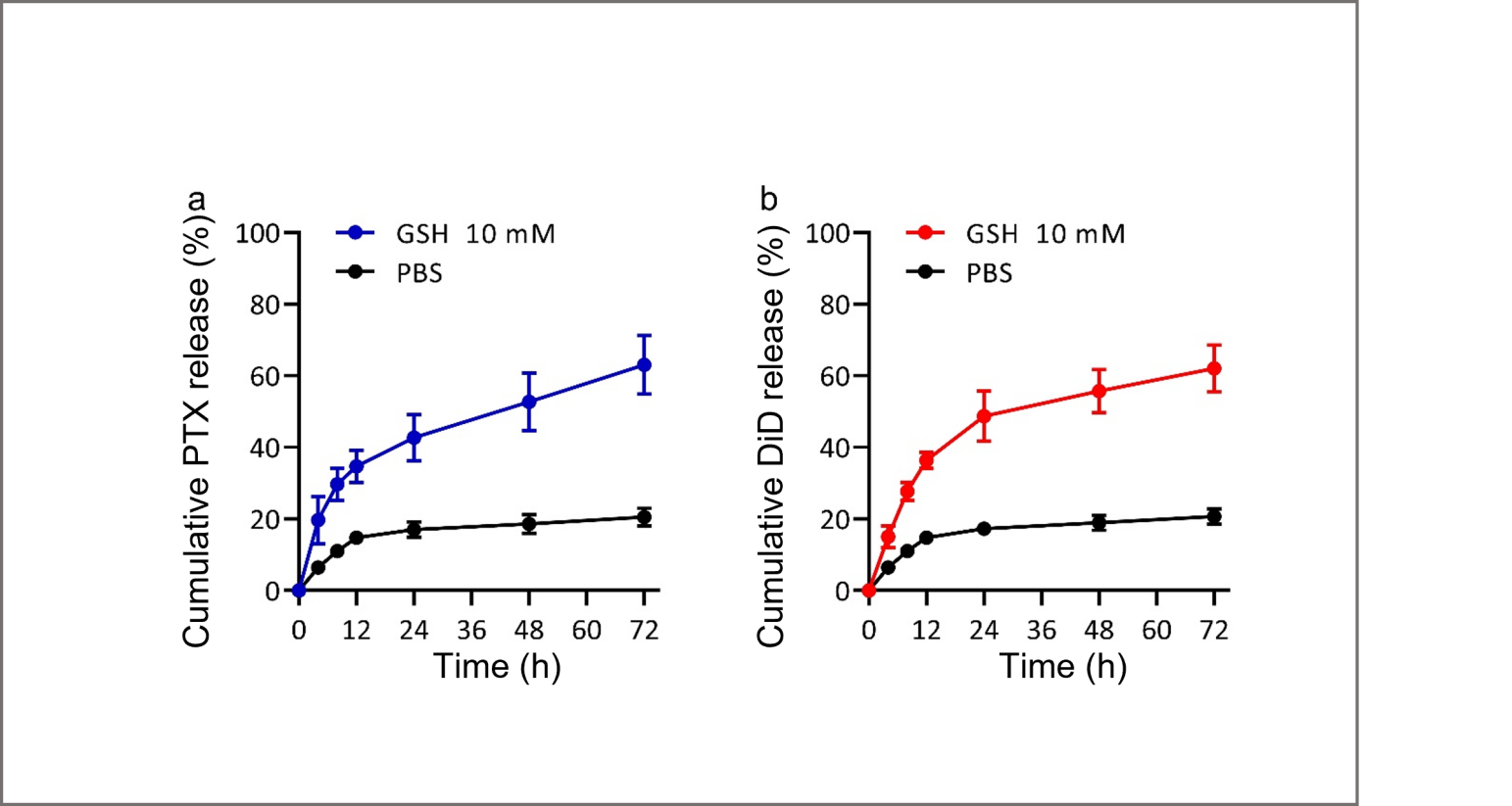


**Figure S2.** The *in vitro* release profile of (**a**) PTX and (**b**) DiD from DCM-[PTX/DiD] NPs with and without GSH over time. Data are presented as mean ± s.d., *n* = 3 independent experiments.

**
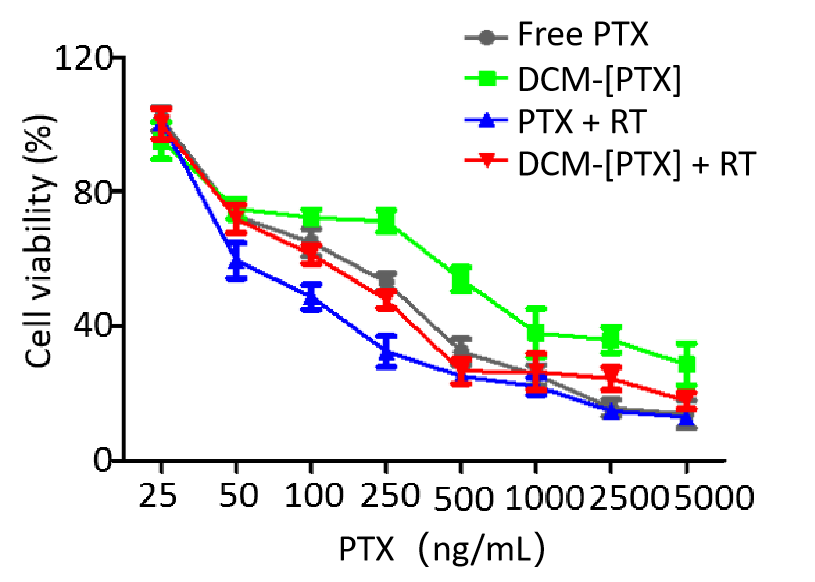
**

**Figure S3.** The synergistic treatment effect between DCM-[PTX] plus radiation for OSC-3 oral cancer cells. PTX or DCM-[PTX] (PTX concentration 0.1 mg/mL) with different dilution were co-culture with cells for 8 h, followed by irradiated with 6 Gy single treatment (X-ray cabinet, USA). After further incubated for 16h, cells were evaluated the relative cell viability by Micro-plate ELISA reader (SpectraMax M2, USA).

**
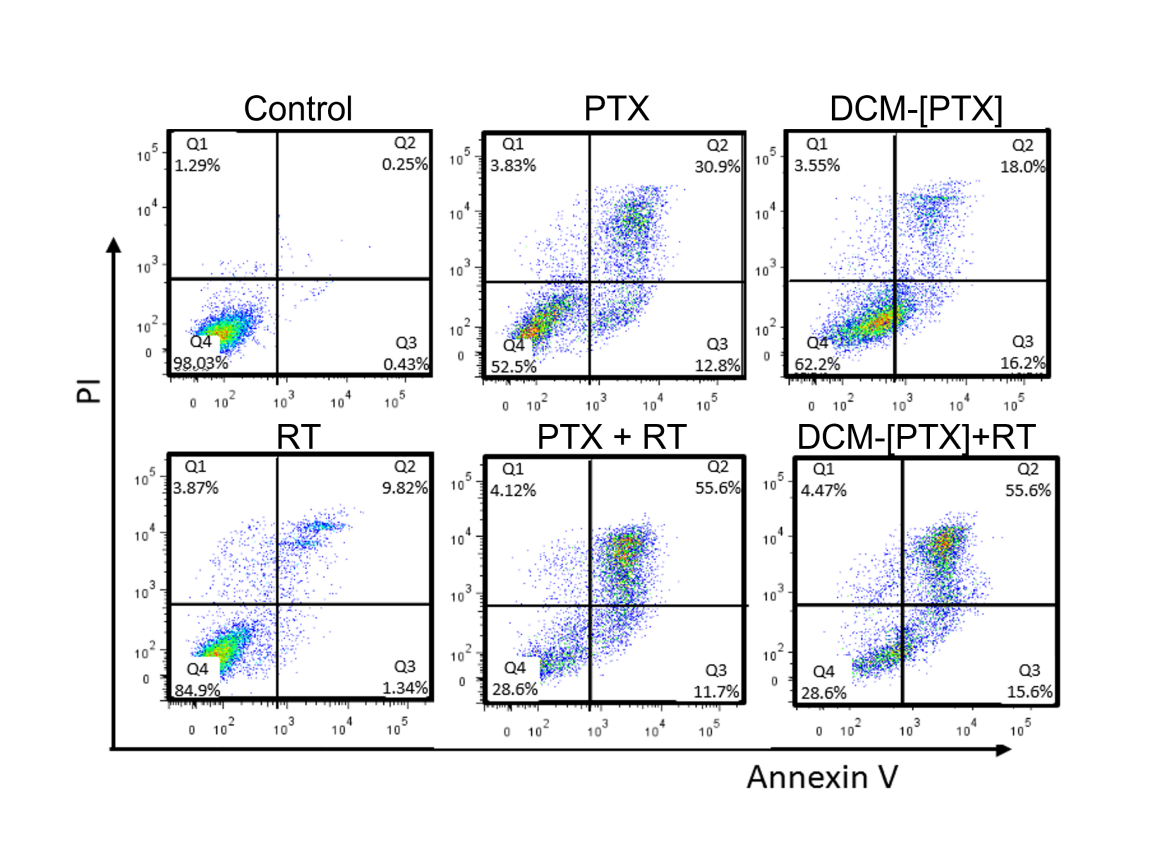
**

**Figure S4.** Detection of the OSC-3 oral cells relative viability by flow cytometry assay using Annexin V-FITC and PI at various cell culture conditions. PTX dose: 0.002 mg/mL; radiation dose: 6 Gy.

**
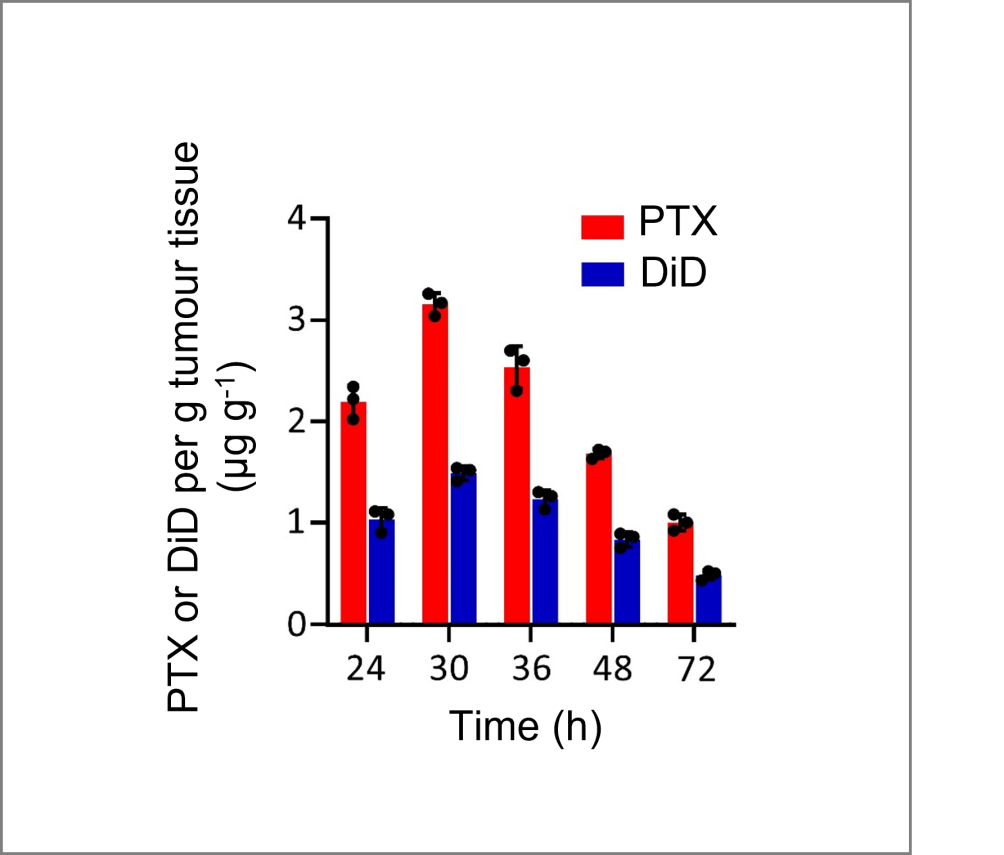
**

**Figure S5.** PTX and DiD distribution retention in tumor tissues at different time points post injection of DCM-[PTX/DiD] NPs. Injection dose of PTX: 5 mg/kg; Injection dose of DiD: 2.5 mg/kg; data were mean ± s.d., *n* = 3 for each time point.

**
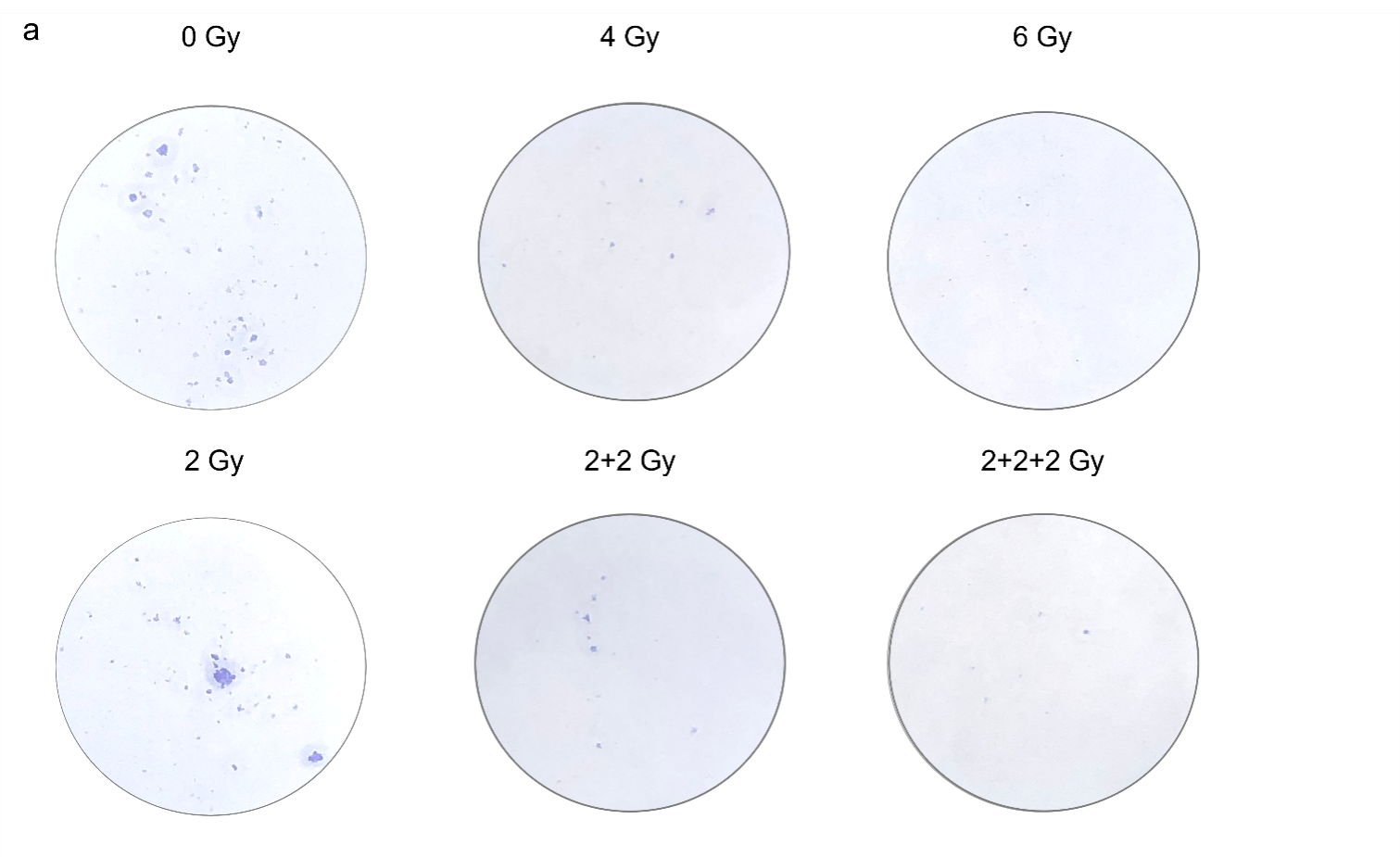
**

**
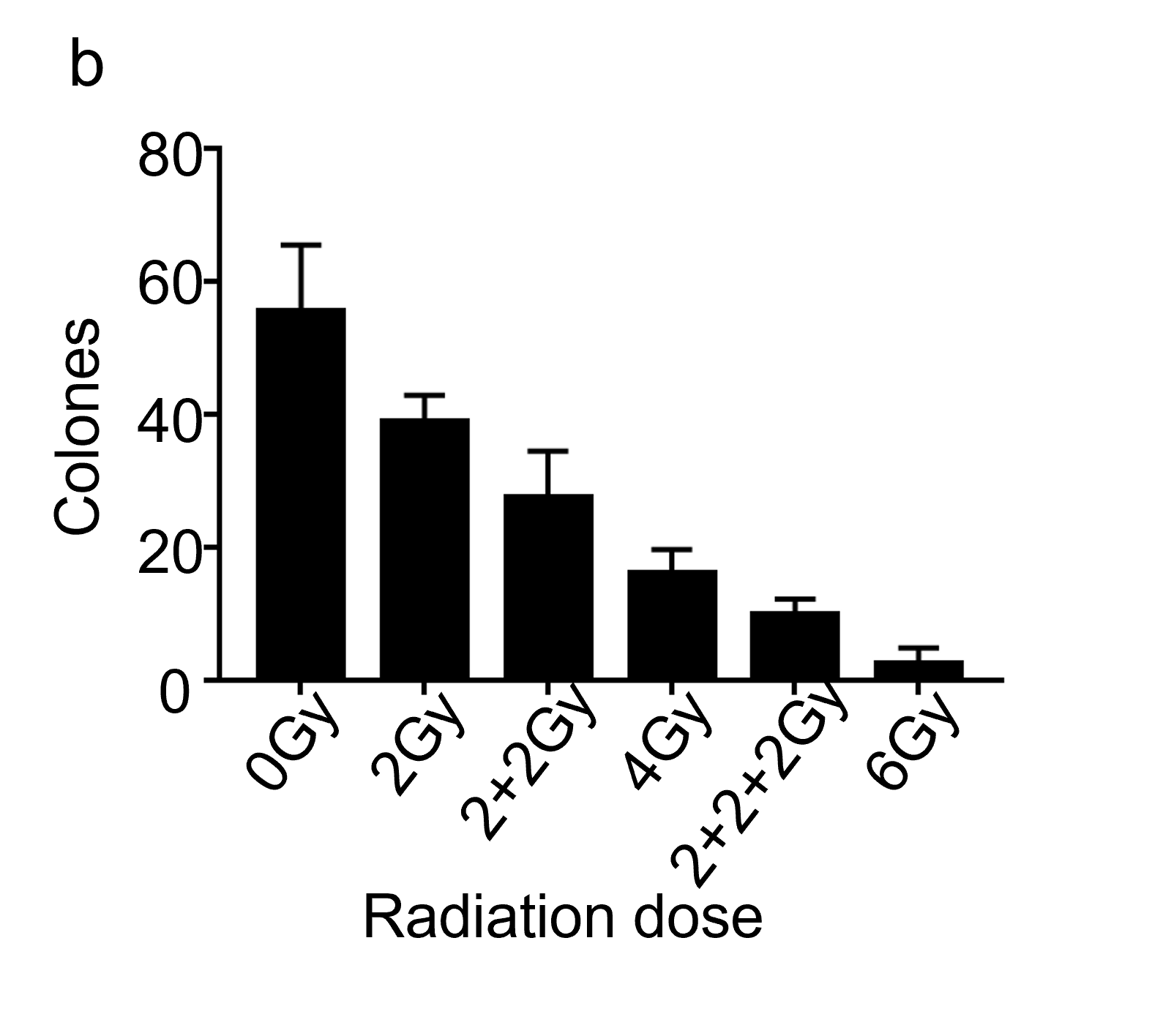
**

**Figure S6. The colony formation assay.** (a) Cells were treated with different radiation doses, including 0 Gy, 2 Gy, 4 Gy, 2 Gy for two times in two consecutive days (total dose 4 Gy), 2 Gy for three times in three consecutive days (total dose 6 Gy), 6 Gy, which have proved the SBRT treatment response superior to the conventional fractionated radiation. (b) The number of colons (defined as more than 50 cells) were counted manually. The independent experiments were repeated for 3 times.

**
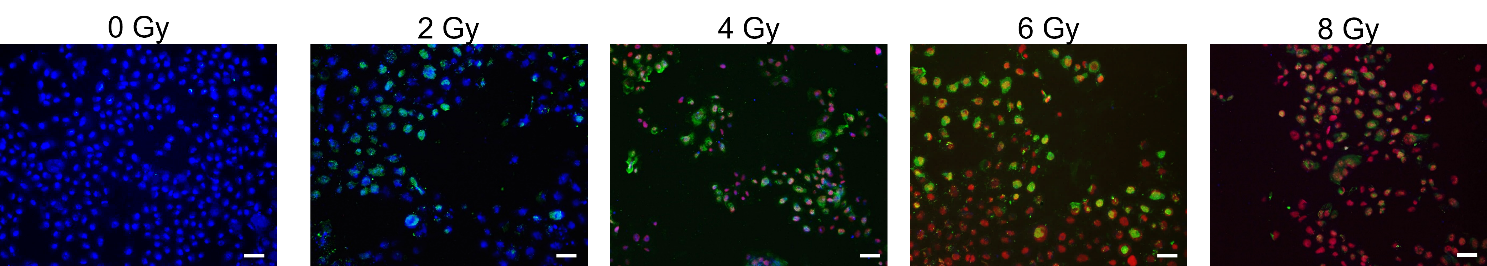
**

**Figure S7.** Assessment of the radiation induced apoptosis or necrosis were detected by CLSM ( Blue: healthy tumor cells, Green: apoptotic tumor cells, Red: necrotic cells, scale bar = 50 µm).

**
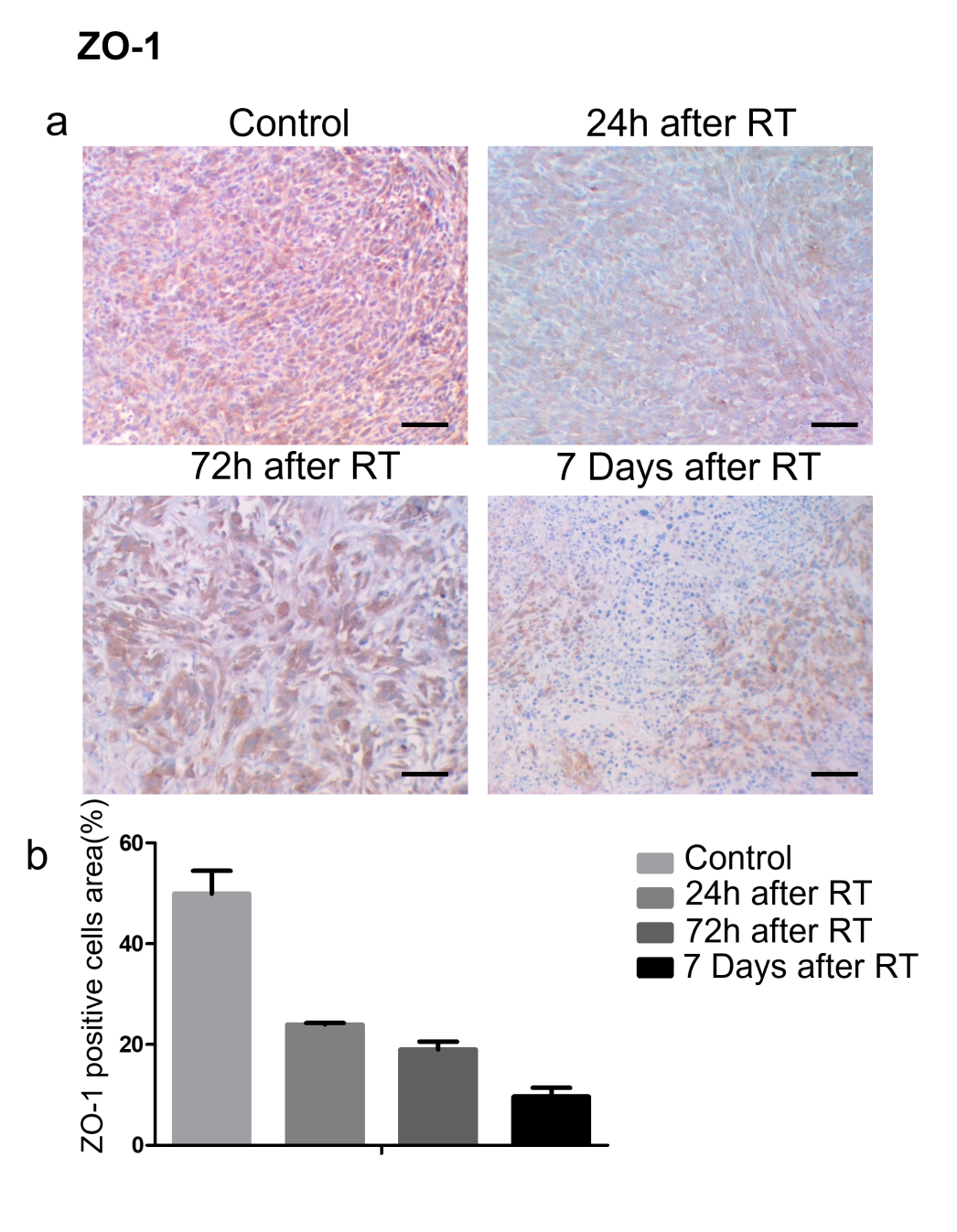
**

**Figure S8. The expression of tight junction protein was significantly decreased after radiotherapy.** (**a**) Nude mice bearing oral cancer xenograft were pretreated single-fraction radiotherapy (12 Gy) and tumor tissues were stained ZO-1 to evaluate the destruction of tight junction between cells (scale bar = 20 µm). (**b**) Quantitative immunohistochemistry for analysis the ZO-1 expression in tumor area by image J software.

**
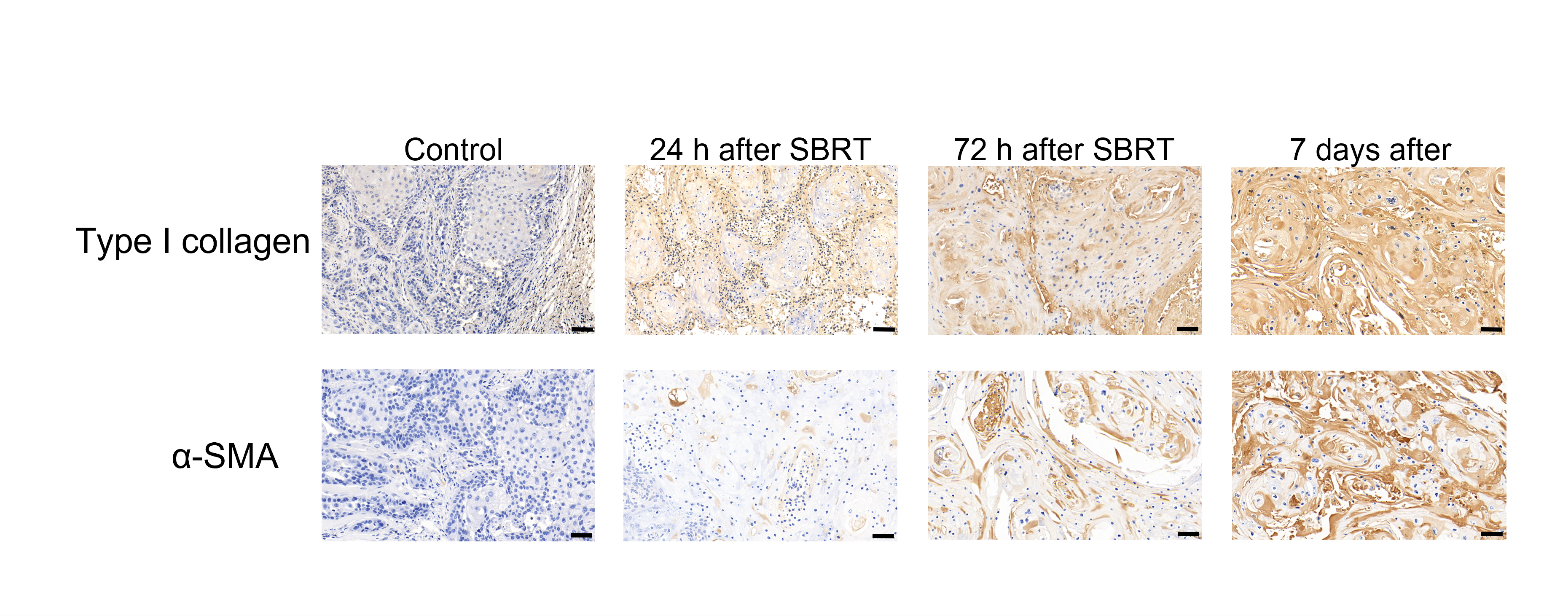
Figure S9.** The expression of stromal tissue markers were detected increase after radiotherapy (scale bar = 50 µm).

**
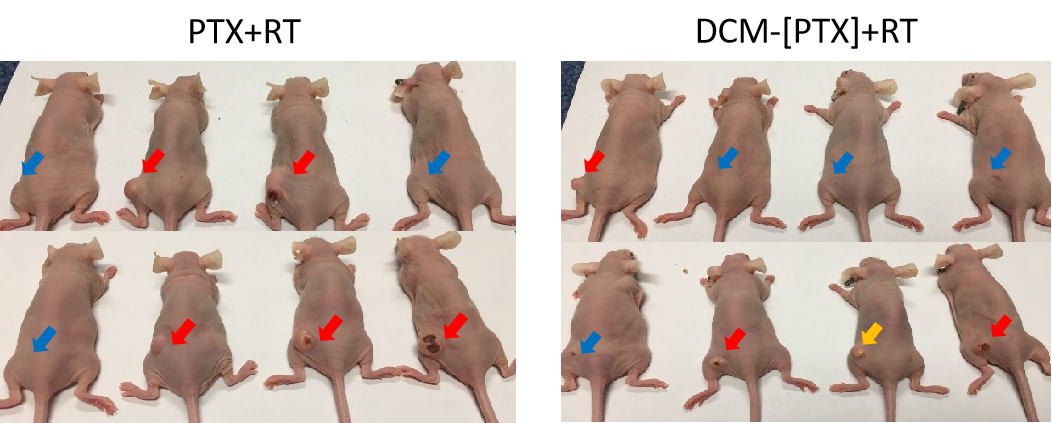
**

**Figure S10.** Representative images of tumor volume in PTX+RT and DCM-[PTX]+RT after treatments in weeks 7. The blue arrow means tumor was totally eliminated; the red arrow means residual small tumor still existed after treatment; the yellow arrow means tumor was disappeared in weeks 8.

**
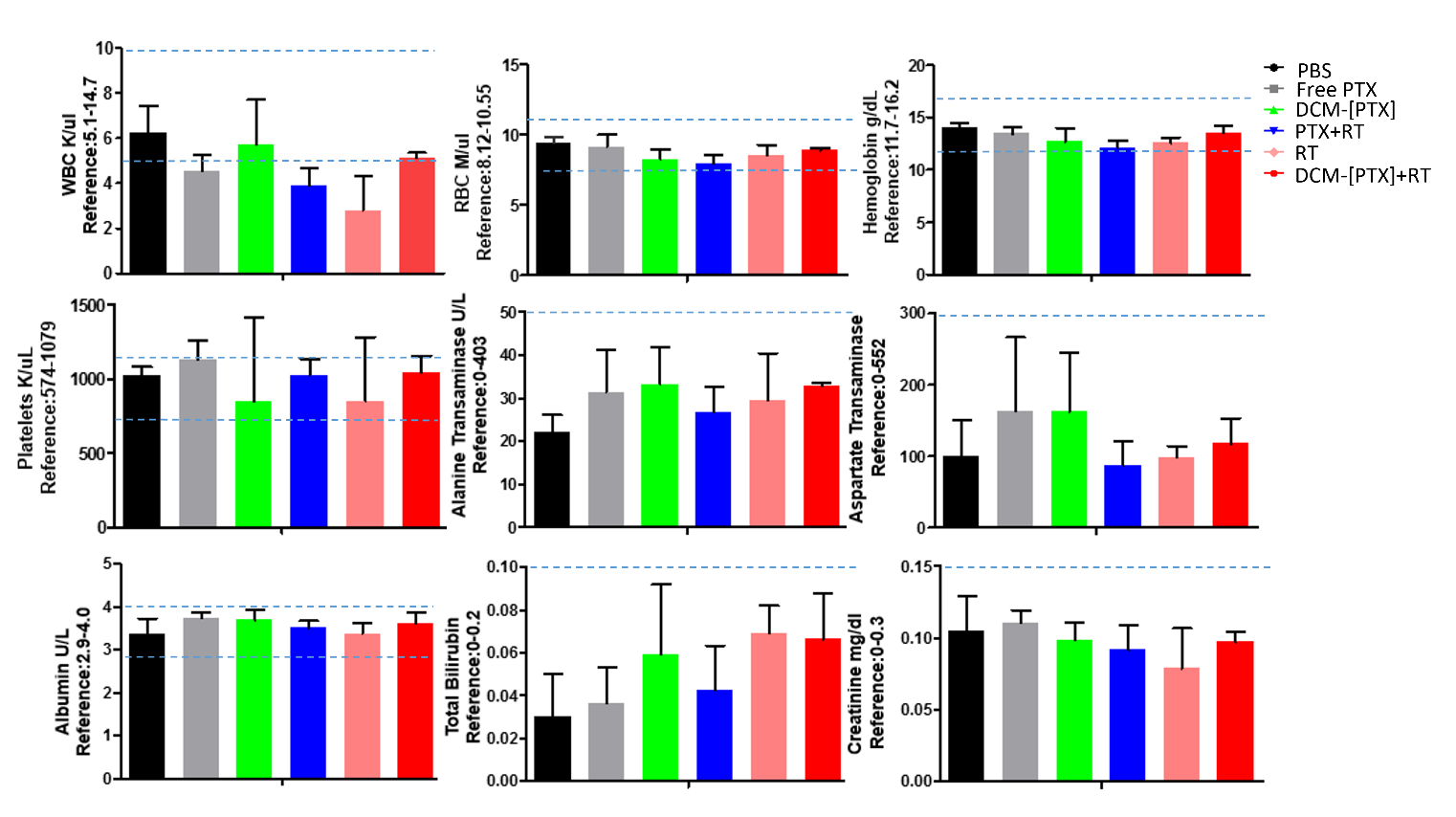
Figure S11.** Blood was drawn after two cycle treatment to test the blood routine in terms of RBC, WBC, platelets, hemoglobin, and liver/kidney function.

**
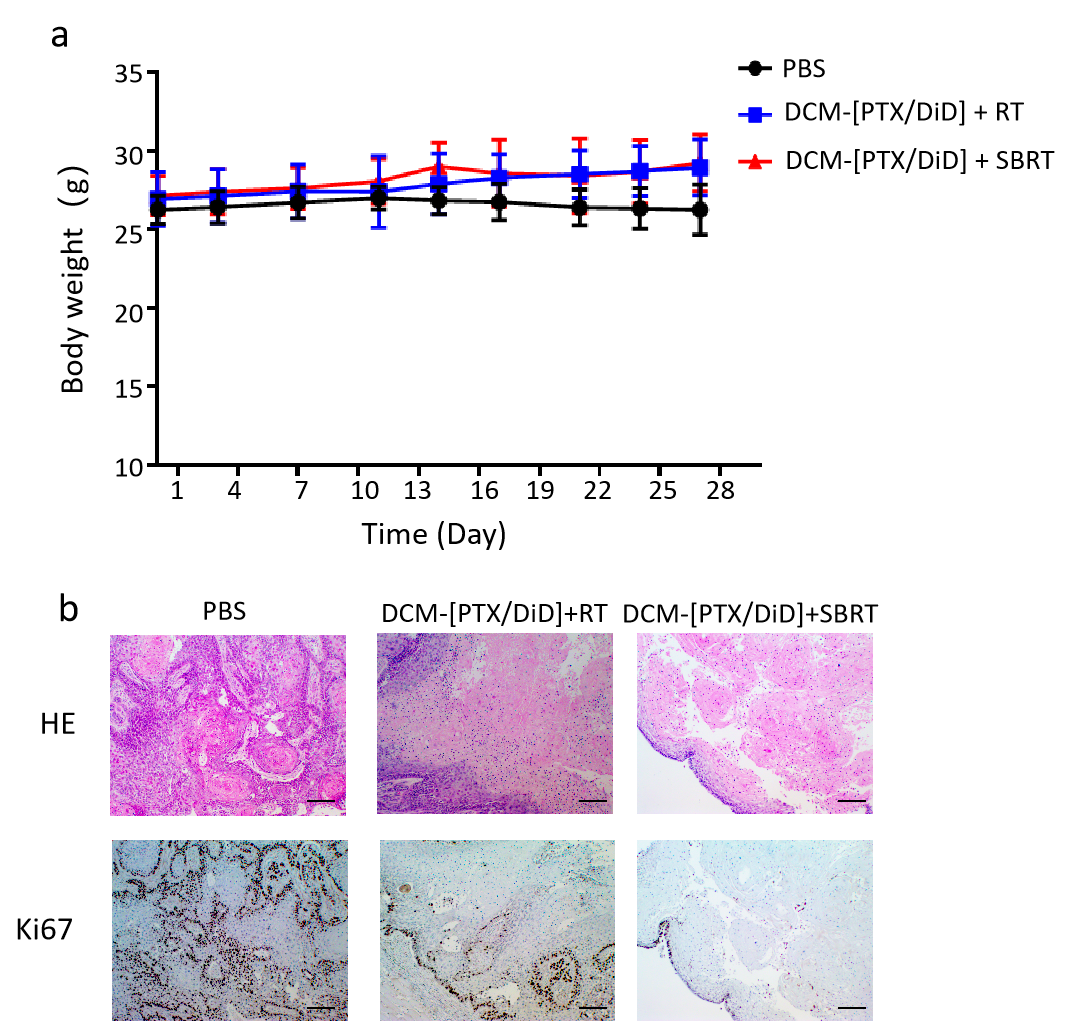
**

**Figure S12. Anti-tumor responds of DCM-[PTX/DiD] NPs in combination with stereotactic body radiotherapy (SBRT) or conventional dose radiation in nude mice bearing OSC-3 oral cancer tumors.** (a) Weight changes of the nude mice. (b) Tumor tissues were collected after treatment to evaluate apoptosis by staining H&E and Ki67 (scale bar = 20 µm).


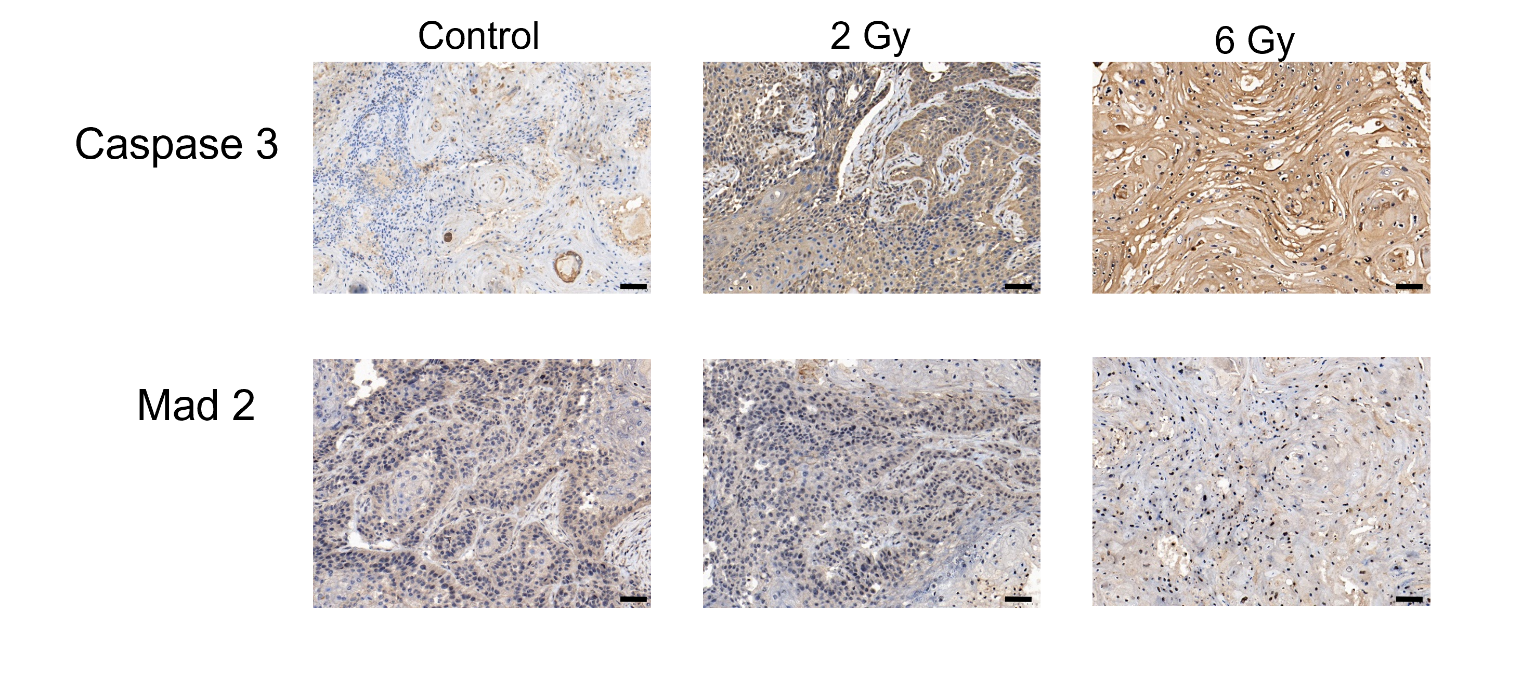


**Figure S13.** Tumor section was stained with Caspase-3 and Mad-2 to evaluate the necrosis and mitotic catastrophe, respectively (scale bar = 50 µm).
